# A comprehensive review on infant formula: nutritional and functional constituents, recent trends in processing and its impact on infants’ gut microbiota

**DOI:** 10.3389/fnut.2023.1194679

**Published:** 2023-06-21

**Authors:** Shiva Bakshi, Vinod Kumar Paswan, Satya Prakash Yadav, Basant Kumar Bhinchhar, Sheela Kharkwal, Hency Rose, Prajasattak Kanetkar, Vishal Kumar, Zakarya Ali Saleh Al-Zamani, Durga Shankar Bunkar

**Affiliations:** ^1^Department of Dairy Science and Food Technology, Institute of Agricultural Sciences, Banaras Hindu University, Varanasi, India; ^2^Department of Livestock Production Management, Sri Karan Narendra Agriculture University, Jobner, India; ^3^Department of Agriculture Economics, Sri Karan Narendra Agriculture University, Jobner, India; ^4^Division of Dairy Technology, ICAR—National Dairy Research Institute, Karnal, India; ^5^Department of Food Technology and Science, Faculty of Agriculture and Veterinary Medicine, Ibb University, Ibb, Yemen

**Keywords:** breastfeeding, milk formulas, infant gut microbiome, non-thermal processing techniques, thermal processing, pediatric nutrition, infant diet formulation, human milk

## Abstract

Human milk is considered the most valuable form of nutrition for infants for their growth, development and function. So far, there are still some cases where feeding human milk is not feasible. As a result, the market for infant formula is widely increasing, and formula feeding become an alternative or substitute for breastfeeding. The nutritional value of the formula can be improved by adding functional bioactive compounds like probiotics, prebiotics, human milk oligosaccharides, vitamins, minerals, taurine, inositol, osteopontin, lactoferrin, gangliosides, carnitine etc. For processing of infant formula, diverse thermal and non-thermal technologies have been employed. Infant formula can be either in powdered form, which requires reconstitution with water or in ready-to-feed liquid form, among which powder form is readily available, shelf-stable and vastly marketed. Infants’ gut microbiota is a complex ecosystem and the nutrient composition of infant formula is recognized to have a lasting effect on it. Likewise, the gut microbiota establishment closely parallels with host immune development and growth. Therefore, it must be contemplated as an important factor for consideration while developing formulas. In this review, we have focused on the formulation and manufacturing of safe and nutritious infant formula equivalent to human milk or aligning with the infant’s needs and its ultimate impact on infants’ gut microbiota.

## 1. Introduction

Nowadays, the major driving forces for the acceptance and consumption of food items on a global scale are their functionality and the health benefits imparted by them. Food production is a foremost and significant driver of environmental changes like biodiversity loss, water pollution and deforestation (1). Alongside, malnutrition in its varied forms involving undernutrition, non-communicable diseases related to diet, and unhealthy diets are the chief contributors to the global burden of several diseases (2, 3). The prevalence of malnutrition heralds a global epidemic that presents difficulties for public health (4). Viewing all such issues, food security and nutrition must be the global political priority (5). Consequently, the development and formulation of health-imparting formulas are gaining more attention from policymakers, researchers, and advocates (6, 7).

Regarding infant nutrition, breastfeeding is a compelling force contributing to a child’s sustainable growth and development. It is a biological first food that is nutritionally optimized, safe for consumption and protects the child against many infections (8). It can reduce stress for both the infant and mother, imparting the child–mother bonding and can literally be called ‘packed with love’ (9). Breastfed children are more likely to achieve full intellectual potential and can ultimately perform better in later life (7). Breastfeeding has advantages for mothers in terms of birth spacing and decreased incidence of breast and ovarian cancer. Furthermore, the environmental cost of breastfeeding is negligible, whereas the economic loss from non-breastfeeding is estimated to be greater than USD 300 billion annually (10, 11). However, some mothers are unable to breastfeed their infants because of medical or physiological reasons like poor mammary gland development (mammary hypoplasia) and hormonal imbalances (12, 13). Nonetheless, promotion and marketing of breastmilk substitutes are thought to be the supplementary hurdle for breastfeeding. Exposure of individuals to this strategic marketing led to reduced breastfeeding initiation and duration, irrespective of the country (7, 14, 15).

Commercial substitutes for breastfeeding milk formulas are consumed worldwide, defining a partial or complete replacement for breast milk to feed young children or infants between 0–36 months of age (16). Milk formulas are distinguished into three main parts, including standard infant formula (IF) (0–6 months), follow-up formula (6–12 months) and toddler formula (13–36 months). Milk formulas are processed food items with a typical formulation of milk proteins, lactose or other sugars, vegetable oils, micronutrients, and some other additives (17, 18). The IFs are for both the small and large populations of mothers who cannot breastfeed their infants. On the other hand, the follow-up or the toddler formulas are superfluous to human needs. These formulas are manifold expensive compared to regular cow or buffalo milk (19, 20). IF is generally available in 3 forms: liquid, powder and ready-to-feed. The powdered form is the least expensive and is mixed with water for feeding. The concentrated liquid form is supposed to be mixed with an equal amount of water. Among all three, the most expensive is the ready-to-feed form which requires no mixing before feeding (21).

In terms of the IF market, there is a broadening of geographical reach and product ranges (7). In 2021, the infant formula market registered a revenue of approximately 38.17 billion USD. The sector is anticipated to expand at a compound annual growth rate (CARG) of over 10% between 2022 to 2030, driven by the increased prevalence of premature birth (22). A few players, including Nestle, Danone, Abbott, FrieslandCampina and Heinz, dominate the IF market. Together, these companies control nearly 60% global IF market share (23). Moreover, the consumption of these infant formulas is rising day by day because of key drivers such as the increasing number of working mothers, rising cases of malnutrition, a concern about infant nutrition and the growing income of the middle class (7).

Undernourished children in poor or undeveloped countries will likely be deficient in foods rich in high-quality proteins comprising the essential amino acids that are the building block of cognitive development and linear growth (24). Milk possesses physical and nutritional characteristics, making it ideal for complementary food. The digestibility-corrected amino acid score of dairy items is higher than other foods. Milk also contains unique plasma insulin-like growth factor 1 (IGF-1), a growth hormone that increases amino acid uptake (25). It is dense in fat, calories, and many micronutrients, like Vitamin A & B_12_ and rich in calcium, phosphorous, magnesium and potassium (26). Milk and its products possess antioxidant potential, and this might be due to the sulfur-containing amino acids (cysteine and methionine), vitamins (A, E), antioxidative enzymes (glutathione peroxidase, superoxide dismutase and catalase) and the appreciable amount of the daidzein, a polyphenolic metabolite (27). All of these play a significant role in numerous functions like immunomodulation, cardiovascular, neural, and metabolic growth and the gut microbiome’s establishment (28). In this paper, we have focused on the several components engaged in the formulation of infant formula, their role in the infant’s gut, the manufacturing process, and non-thermal technologies utilized to preserve nutritional value and extension of shelf-life.

## 2. Choice of milk or milk substitute for IF formulations

The chemical nature of human milk is complex. The general composition involves 87–88% water, 1.0% protein, 3.8% fat and about 7% lactose (29). Lactose and fat majorly contribute to total energy (30). Although its composition is very dynamic and varies over time according to the needs of a growing child, the lactose content remains fairly constant (after 21 days of parturition). Another function of lactose is maintaining constant osmotic pressure and aiding in mineral absorption (21). Human milk possesses two classes of protein, namely whey and casein. The whey remains liquid in an infant’s stomach, making it easier to digest, while the casein forms clots or curds. The major whey proteins include lactoferrin, alpha-lactalbumin and secretary IgA. Other proteins include folate-binding protein, lipase, amylase, lysozyme, Bifidus factor, anti-chymotrypsin and alpha-1-antitrypsin, and haptocorrin (31). In terms of vitamins and minerals, human milk possesses an adequate amount to support the normal growth of an infant, except vitamins K and D. The role of minerals is their contribution in various physiological functions and formation of essential amino acids and are biologically crucial to structural and catalytic molecules (21).

Besides human milk, bovine milk is also a good source of fats and lipids, proteins, carbohydrates, minerals, and vitamins. The protein and mineral content of bovine milk is higher when compared to human milk. Also, the lactose content of human milk is approximately 7 percent [involving 1% of oligosaccharides (OSs)], while bovine milk has a 4.5% lactose content. In contrast, the amount of whey protein in both kinds of milk is approximately similar (32). The casein in bovine milk is eight folds higher if compared to human milk. All such differences should be considered while formulating IF. Also, the whey-to-casein ratio (20, 80) of bovine milk should be modified in such a way that it mimics the mature human milk’s whey-to-casein ratio, i.e., 60:40 (33). For a quick illustration, a brief mention of the nutritional composition of milk from different milk species is presented in Table 1. Furthermore, mare, camel and llama milks have a higher Ig (Ig) content than cow, goat, sheep, and human milk. Donkey milk is comparable to human milk as it contains lower casein and higher serum protein. Even the lysozyme level of donkey milk is two times higher than human milk, corresponding to its higher antimicrobial activity. Moreover, human milk and mare’s milk contain less saturated fatty acids than the milks of other species (34). Lipid classes in selected milks include cerebrosides in camel, deer & buffalo; gangliosides in deer, sheep & camel; and plasmalogens in buffalo, deer & goat (35).

**Table 1:** Nutritional composition of milk from different species used in infant formula formulation.

Parameter	Cow	Buffalo	Sheep	Goat	Camel	Donkey	Human	References
Total solids (g/L)	118–130	157–17.2	181–200	119–163	119–150	88–117	107–129	(42)
Fat (g/L)	33–54	53–90	50–90	30–72	20–60	03–18	21–40	
Ash (g/L)	07–08	08–09	08–10	07–09	06.9–09	03–05	02–03	
Lactose (g/L)	44–56	32–49	41–59	32–50	35–51	58–74	63–70	
Protein (g/L)	30–39	2.7–4.7	4.5–7.0	3.0–5.2	2.4–4.2	1.4–2.0	0.9–1.9	
Total casein (g/L)	24.6–28	32–40	41.8–52.6	23.3–46.3	22.1–26.0	6.4–10.3	2.4–4.2	
Total whey protein (g/L)	5.5–7.0	6	10.2–16.1	3.7–7.0	5.9–8.1	4.9–8.0	6.2–8.3	
SFA (%)	52–76	62–79	47–80	57–78	24–70	44–68	35–57	(43)
MUFA (%)	18–34	23–30	13–30	10–29	14–44	15–35	17–45	
PUFA (%)	2–6	2–5	2.4–9	0.5–8	2–6	11–20	10–31	
Lactoferrin (g/L)	0.02–0.5	0.02–0.3	0.7–0.9	0.02–0.3	0.2–0.9	0.3	0.7–1.7	
Immunoglobulins (g/L)	0.15–1.0	0.5–1.3	0.5–0.7	0.15–0.5	0.55–0.8	1.3	0.6–1.8	
Energy (kJ/L)	2,709–2,843	4,244–4,779	4,038–4,439	2,802–2,894	2,410–3,286	1,607–1803	2,516–3,245	(44, 45)

Bovine milk is used as a primary base material for the formulation of infant formulas because of its higher volume production, well-established chain distributions, and many recognized functional attributes of its components (36). Nonetheless, sometimes infants suffer from cow’s milk allergies (CMA), which is an immune-mediated reaction. Here IgE antibodies bind to the surface of mast cells, and subsequent exposure leads to degranulation of mast cells and release of mediators, including histamine & leukotrienes. All this causes symptoms involving throat tightness, urticaria, angioedema, abdominal pain, diarrhea, vomiting and dizziness (37, 38). In such cases, camel, donkey milk-based IF can be an alternative option. Human and donkey milk has a similar composition as well as antigenic and protein homogeneity (39). Camel milk-based IF has superior anti-inflammatory activity, so this can be a practical option in hypo-allergic IF production (40). Further, other reasons for using alternative formulas can be the disorders related to carbohydrate metabolisms, like deficiency of primary lactase, galactosemia or adopting a vegan lifestyle. However, non-dairy formulas like soy-based formulas can also be given in these cases. At the same time, protein hydrolysates are also developed for infants suffering from soy and milk protein intolerance (33). Consequently, developing newer infant formulas and improving existing ones by enhancing functional properties is a task for researchers and scientists and can be achieved by using milk from different species (41).

### 2.1. Bioactive peptides in milk

The milk proteins are the primary source of bioactive peptides, which are short-chained sequences of the amino acids and may be released through enzymatic action or *in vivo* fermentation with starter (lactic acid bacteria) (46). These bioactive peptides are generally small in size and range from 2–50 amino acid residues, and these can be used in the manufacturing of infant formulas (47). In general, Igs, α-LA, β-LG, protease-peptide fractions, lactoferrin, caseins and a minimal amount of whey proteins like transferrin and serum albumin are the major fractions of the proteins present inside bovine milk. These peptides are formed *in-vivo* by the gastrointestinal (GI) processes. These can also be produced *in vitro* by enzymatic hydrolysis, encoded in precursor sequences of the native protein. The peptides are later purified by various separation techniques like ultrafiltration, size exclusion, reversed-phased high-performance liquid and ion exchange chromatography (28, 48).

Several bioactive compounds in bovine and caprine milk are effective against cardiovascular, digestive, neurological, immunological and endocrine system disorders, displaying various functional properties like antithrombotic, anti-hypertensive, anti-microbial, anti-oxidant, immunomodulatory and anti-hypertensive activities (49, 50). For example, α- Lactalbumin is broken down into smaller peptides depicting antibacterial, immunostimulatory and prebiotic properties. It also improves the absorption of minerals (51). Parastouei et al. (52) assessed the content of bioactive peptides in milk from different species, including cow, buffalo, goat, camel, sheep, donkey horse and humans. The lowest and highest concentrations of the total peptides resistant to digestion were found in human and sheep’s milk. The casein of donkey milk contained a higher inhibitory peptide of dipeptidyl peptidase IV (DPP- IV) & DPP- III, and angiotensin-converting enzyme (ACE). However, whey protein in camel milk contains inflated ACE-inhibitory peptides. Kumar et al. (53) employed commercial proteases *viz.* alcalase, papain and chymotrypsin for hydrolyzation of casein fraction in camel milk protein. It was established that the hydrolysate derived possessed higher antioxidant potential and antimicrobial activity.

**Figure 1:**
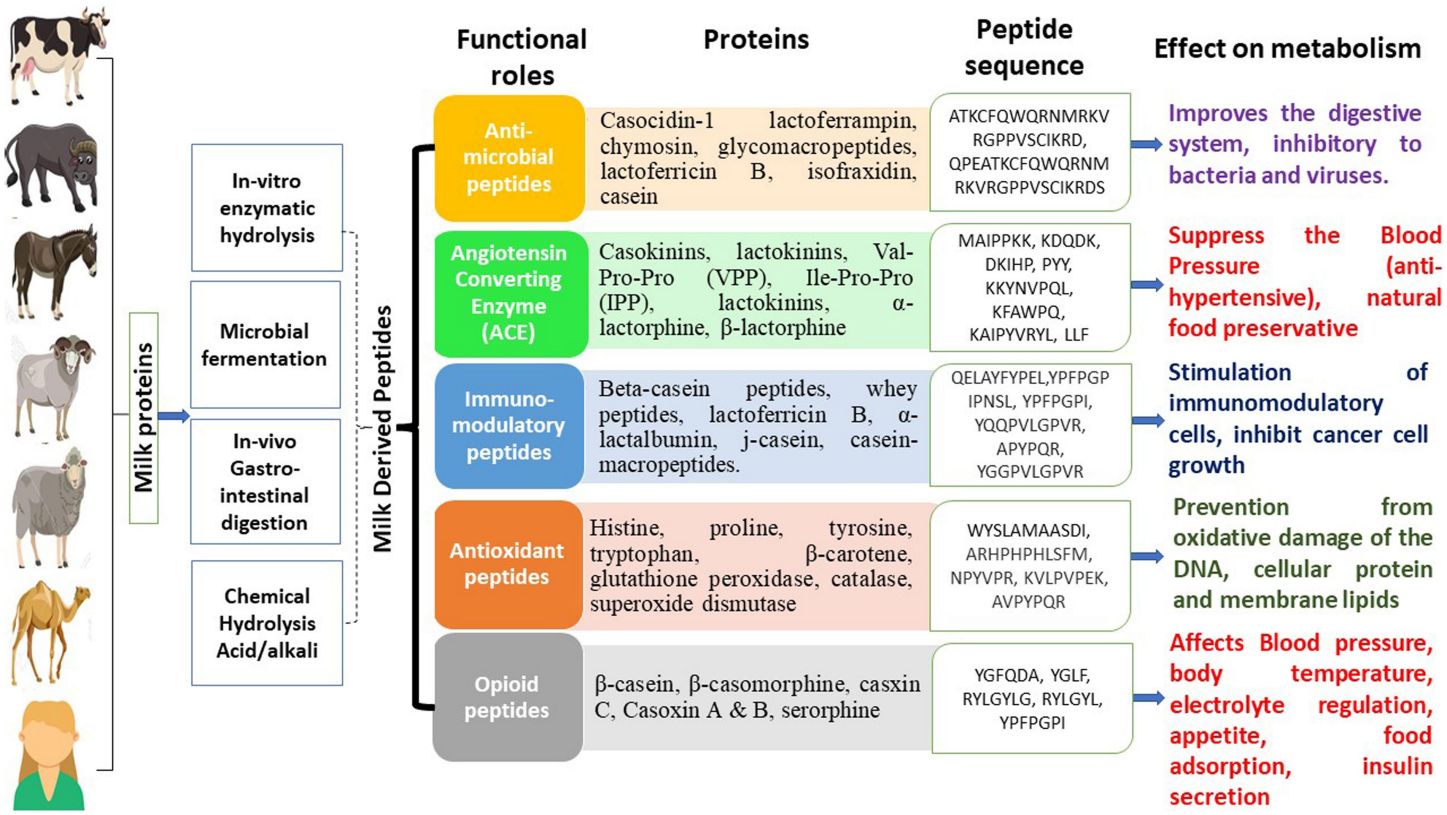
Bioactive peptides in milk and their functional effects.

In another investigative work, Chen et al. (54) utilized *Lactobacillus plantarum* to ferment goat milk and later evaluated its ability to generate ACE-inhibitory peptides. The produced hydrolysate showed an ACE-inhibitory activity of 88.91%, and after purification by RP-HPLC and ultrafiltration, it displayed 91.62% ACE inhibitory activity. Pei et al. (55) hydrolyzed yak milk by the action of pepsin, and the hydrolysate generated depicted antimicrobial activity against *Salmonella paratyphi*, *E. coli*, *Enterobacter cloacae* and *Listeria innocua*. Moreover, the glycol-macropeptide of sheep casein has been illustrated to have antiplatelet aggregation activity, and this property increased further with hydrolysis by trypsin. Thus, the increasing interest of researchers in bioactive peptides has lately been augmented exploration of milk bioactive peptides from other species (47). Nowadays, some of these novel components isolated from bovine and caprine milk are commercially available. The important consideration is that while adding these bioactive peptides to IF, lowering the formula’s overall protein concentration is crucial (21). Figure 1 illustrates various classes of bio-peptides derived from milk, along with their functional roles in the health of mankind. For more details about bioactive peptides in milk, readers may refer to Mohanty et al. (46), Guha et al. (47), Nielsen et al. (56) and Park and Nam (57).

## 3. Formulation of infant formula: ingredients and nutrients

Infant Formulas are developed in a way to maintain a balance of macro and microelements. Typical ingredients for formulating IF are carbohydrates, fats, proteins, vitamins and minerals (33). The ingredients [whey protein isolate (WPI), skim milk powders (SMP), whey protein concentrate (WPC) etc.] made from milk are generally used as a protein source. Mainly vegetable fats are used as the fat source, but animal milk fats can also replace them (58, 59). No matter where the macro and micro elements are isolated, they still affect the physiological properties of the IF to be developed. Numerous clinical trials have been conducted to determine the efficacy of these elements in the formulation of IF; some of them are mentioned in Table 2. Various components and their effect on IF are discussed well in the subsequent sections.

**Table 2:** Clinical research conducted on the inclusion of various components in formulations of infant formula.

Components	Study conducted on	Interventions	Feeding duration	Major outcomes	References
Experimental animals					
Whey & β- casein	New-born female rhesus monkey (*n* = 30)	Free amino acids (taurine, alanine, glutamine & glutamate) in isocaloric but regular protein (2.1 g /100 kcal) and reduced protein (1.8 g /100 kcal) IFs.	Until 4 months of age.	By adding free amino acids in IF, the infant’s metabolic & growth performance improved.	(190)
Whey & β-casein	Crossbreed piglets (3 days old) (*n* = 32)	Formulations: only whey protein, whey + casein	Feeding period: 2–4 weeks.	Whey + casein showed improved growth performance & immune regulatory balance compared to formulation 1.	(191)
PDX/GOS & MFGM	Rats (8 weeks of age)	Three formulations were made; 1st. GOS 20.86 g/Kg + PDX 6.44 g/Kg; 2^nd^ WPC + 15.9 g/Kg MFGM, 3^rd^ GOS/PDX & MFGM.	Diets were given from the 21^st^ postnatal day	The beneficial gut microbiota significantly improved by PDX & MFGM blend. The visceral hypersensitivity induced by maternal separation was ameliorated.	(192)
PDX/GOS	New-born piglets (2-day age) (*n* = 25)	Milk replacer consisting of 2 g/L of PDX/GOS	Up to 33-day age	PDX/GOS ingestion during early life enhances the recognition memory.	(193)
Osteopontin	Crossbred piglets (*n* = 48)	The bovine milk is fortified with vitamins & minerals (15 g/L), electrolytes (6 g/L) and osteopontin (319 mg/L).	Feeding period: 19 days	Osteopontin supplementation above basal levels of bovine milk induced minor gut structure improvement & systemic immunity without impacting cognitive function.	(194)
Newborns Infants					
α-Lactalbumin & LC-PUFA	Healthy term infants (*n* = 259)	Protein-reduced formula (1.89 g/100 g) consisting of α-lactalbumin enriched whey & LC-PUFA	Until 4 months of age	The findings revealed no evidence of long-term impacts of early food on anthropometry evaluated at 4 years of age.	(195)
α-Lactalbumin	Healthy full-term infants (≤40 age) (*n* = 308)	1^st^ IF with 1.0 g/dL protein (bovine α-lactalbumin), 2^nd^ & 3^rd^ formula with 1.3 and 1.5 g/dL protein, respectively.	Until 4 months of age	During 1–4 months of age, the IF with 1.0 g/dL protein promotes weight gain and growth, similar to infants that were exclusively breastfed.	(196)
DHA/ARA	Healthy infants (≤9 days old) (*n* = 91)	Formula consisting of 0.64% of total fatty acids as ARA and either 0.32, 0.64, or 0.96% of total fatty acids as DHA.	During infancy	Supplementation of IF with ARA & DHA in the first year of an infant’s life has a protective effect against allergy and also delays allergy in early childhood.	(197)
DHA/ARA	Infants (< 60 days of age) (*n* = 233)	Infant & the follow-on formulas containing 34 mg of ARA/100 kcal and 17 mg of DHA/100 kcal.	Received IF for 4 months of age	Infants had a lower incidence of nasal congestion, bronchitis/ bronchiolitis, cough, and diarrhea.	(198)
DHA/ARA	Infants (<21 days of age) (*n* = 89)	A formula containing 0, 25, or 34 mg ARA and 17 mg DHA /100 kcal.	Feeding period: 10 weeks	ARA may have an immune-regulatory effect by reducing B cell activation markers & their subsequent function.	(199)
MFGM	Infants (<2 months) (*n* = 160)	The energy density reduced from 66 to 60 kcal/100 mL; protein from 1.27 to 1.20 g/100 mL, and supplementation with MFGM-enriched WPC (4% w/w)	Formula feeding until 6 months of age	MFGM supplementation reduces acute otitis media risk. Humoral response to *Pneumococcus* vaccine is immunomodulated.	(200)
Carotenoids	Preterm (<33 weeks gestational age) (*n* = 203)	Iron-fortified, ready-to-feed liquid IF supplemented with lutein/ zeaxanthin, β-carotene & lycopene carotenoids at concentration of 211, 219, and 143 μg/L, respectively.	Until 40 weeks post-menstrual age	The plasma carotenoid concentration was higher with supplementation and was similar to that of human milk-fed infants. The supplemented group also displayed increased sensitivity of the rod photoreceptors.	(201)
Pre & Pro-biotics, MFGM, PUFA	Infants (0–2 months) (*n* = 170)	Supplementation with MFGM [10% of the total protein content (w/w)], symbiotic (FOS & inulin 1:1), *Bifidobacterium infantis* & *Lactobacillus rhamnosus*, LC-PUFAs (ARA & DHA)	Feeding during the first 18 months of life	Compared to breastfeeding, experimental IF had a similar impact on growth patterns over the first 18 months of life. Infants fed with experimental formula showed improved brain maturation which was assessed as visual function.	(202)
*Bifidobacterium lactis*	Infants (≤14 days of age) (*n* = 32)	IF with probiotic strain *Bifidobacterium lactis*, @ 1 × 10^6^ CFU/g.	Up to 4 months of age	Probiotic IF intervention was well-tolerated and safe and reflected a healthy early pattern of weight gain.	(203)
Pre & Postbiotics	Infants (0–4 weeks of age) (*n* = 90)	The concentration of glucose, lactose, prebiotics & fiber 0.3, 7.1, 0.8 & 0.6 g/100 mL. The postbiotics concentration was 30% of dry wt. & contained 3′-galactosyllactoses.	Until 17 weeks of age	Specific prebiotics & postbiotics in IF may cause changes in intestinal microbiota composition, bringing the resulting fecal metabolite profile of IF-fed infants closer to that of breast-fed ones.	(204)
LF	Healthy children (12–32 months) (*n* = 3,847)	LF-fortified growing-up formula.	Feeding period: 13 weeks	Intake of LF reduced the frequency of acute GI symptoms in children. The total number of days having acute respiratory symptoms was significantly lesser in the LF group.	(205)
LF	Infants (4–6 months of age) (*n* = 260)	LF-fortified (38 mg/100 g) formula milk	Feeding period: 3 months	Reduced incidence of cough, running nose & wheezing in infants in both fortified IF & breastfed infants.	(206)
LF-MFGM	Infants (10–14 days of age) (*n* = 451)	Bovine MFGM and whey protein-lipid concentrate @ 5 g/L and bovine lactoferrin @ 0.6 g/L	Up to 180 days of age	The neurodevelopmental profile of infants was accelerated at day 365 & the language category improved at day 545. At 545 days of age, fewer events of diarrhea & respiratory system diseases.	(207)
Osteopontin	Healthy infants (<1 month of age) (*n* = 240)	Whey-adjusted formula milk used as a base & fortified with bovine osteopontin @ 65 mg/L or 130 mg /L.	Up to 6 months of age	Osteopontin addition to IF, alters cytokine responses & metabolism of amino acids, making them more similar to breastfed infants. The lower occurrence of pyrexia.	(208)
Choline & DHA	Preterm infants (<32-week post-menstrual age) (*n* = 24)	Enteral nutrition is given @ 30 mg/kg/day choline and 10 mg/kg/day DHA.	Feeding period: 10 days	In combination with DHA, choline increases DHA-phosphatidylcholine more than DHA alone, which might improve its supply to the brain and eyes.	(209)
Ganglioside	Infants (8 weeks of age) (*n* = 91)	Addition of milk-derived lipid to a standard IF to increase glycoside level by 2–3 mg/100 g.	Until the age of 24 weeks	IF supplementation with complex lipids appears to be beneficial for cognitive development in healthy infants	(210)
Carnitine	Low-birth weight infants (<1 week old) (*n* = 25)	Infants fed with premature IF containing 10 mg/100 g carnitine	Feeding period: 9 days	Carnitine supplementation in IF increased its concentration in serum and improved lipid metabolism.	(211)

### 3.1. Carbohydrates

There are two broad categorizations of carbohydrates: non-glycemic and glycemic carbohydrates. The glycemic ones are absorbed and digested in the small intestine and are later followed by increased blood sugar levels. Whereas non-glycemic pass undigested to the large intestine and does not lead to an increase in blood glucose, hence exerting prebiotic effects (60). The EU legislation for the IF specifies the minimum content of the total carbohydrates, which is 9 g/100 kcal (61). The glycemic carbohydrates act as the potential energy source for infants (62). Regarding the bovine and caprine milk-based IFs, lactose is the primary source, consisting of approximately 7–7.5 g/100 mL lactose content. Pure lactose is available from the processing of whey and bovine milk, which is subsequently used for the fortification of IF for matching human milk lactose levels (45). On the contrary, some other sources of carbohydrates are also permitted in IF, like sucrose, maltose, and glucose. These are generally permitted in IF, which are made from protein hydrolysates and are helpful in palatability improvement as they can be bitter (36, 61, 63). The maltodextrin incorporation in food powder has improved properties like resistance to caking and crystallization, reducing Maillard browning, lowering bulk density, and enhancing stability and dispersibility (64).

Non-glycemic carbohydrates involve the OSs present in milk (45). The Human milk OSs (HMOS) are resisted for digestion in the upper GI tract and show the prebiotic effect by acting as a substrate for the growth of many beneficial bacteria like *bifidobacteria* (65). They can also serve as decoys that prevent toxins and pathogenic bacteria from binding to the target epithelial cells, showing antibacterial activity (66). The composition of different milk sources has already been presented in Table 1. Besides, the concentration of HMOSs is 5–20 g/L, i.e., higher than the milk of other domesticated animals, e.g., bovine 30–60 mg/L, sheep 20–40 mg/L, goat 60–350 mg/L and donkey 250–300 mg/L, respectively (67, 68). The bovine colostrum (day 0) contains more 6′-sialyllactose, disialyllacto-N-tetraose, and LS-tetrasaccharide. Subsequently, their concentration decreases as milk matures (days 3–5) (69). Martin-Ortiz et al. (70) demonstrated that caprine milk OSs decreased with lactation stage like at 488 mg/L (day 1) to 112 mg/L (day 120). The sialylation is higher in colostrum (>80%) but later decreases to approximately 40% on day 120 (71). Likewise in donkey milk lower sialylated OSs were observed at 15th day of lactation (72).

Moreover, the biosynthetic pathways of HMOSs are considerably different from that of free bovine milk OSs (73). The neutral OSs are the predominant ones in human milk, while these are merely present in bovine and caprine milk. Although bovine milk contains fewer OS structures than human milk, these two shares at least ten common OS structures, including the acidic 6′-sialyllactose and 3′-sialyllactose (74). Contrary to that, porcine milk has a higher concentration of neutral OSs (75). Shi et al. (76) reported that camel milk OSs were most similar to human milk compared to cow, goat, camel and sheep milk OSs. Furthermore, the structure of caprine milk OSs is more similar to human milk OSs (45, 66). Lacto-N-biose unit, regarded as a building block of type 1 HMOS, is also found in goat milk OSs. Again, the presence of neutral structures (galactosyl-lactose & lacto-N-hexaose) and sialylated structures (3-6-sialyl-lactose & disialyl-lactose) in human and goat milk OSs, explains the similarities among them (77). Only three acidic OSs in donkey milk have been quantified: 3-sialyllactose, disialyl-lacto-N-tetraose and 6-sialyllactose. But they are present in lower concentrations than in human milk (78). Leong et al. (79) suggested that OSs naturally present in IF based on goats’ milk exhibit strong anti-pathogen adhesion and prebiotic properties and might enhance newborns’ gut health.

### 3.2. Proteins

The IF composition is strictly regulated by various guidelines set up by governing bodies like the Food and Drug Administration (FDA) in the USA, the EU in Europe and internationally, and the Codex Alimentarius Commission (80). The milk-based IF is formulated in such a way as to contain 1.3–1.5 g/100 mL protein approximately. It is still higher than human milk to compensate for the amount of essential amino acids (81). The L-form of amino acids is only permitted to be added, whereas D-form is not allowed because it can cause D-lactic acidosis (21). The high protein also includes a faster weight gain in infancy and has been later correlated to obesity. Thus, a lower protein intake (1.8 g/100 kcal in IF) is suggested to stimulate health effects for longer period of time (65). In the case of bovine milk, protein content is generally achieved by combining WPC, WPI and SMP (36). Although, for caprine milk based IF, whole milk is used as a base to achieve the required protein concentration (82). Additionally, partially or fully hydrolyzed whey proteins and demineralized powdered whey or hydrolyzed demineralized powdered whey are also utilized to produce IF (83–85). Infants allergic to soy and bovine protein hydrolysates are given the amino acids-based formulas (86).

The protein quality of the IF is its ability to fulfill the metabolic requirements for amino acids and nitrogen. The determination of protein quality is generally assessed by protein-digestibility-corrected amino acid scores (PDCAAS) (87). This method has some limitations, such as the values of PDCAAS are calculated on the fecal digestibility basis of the crude protein, and the digestibility of amino acids is determined accurately at the ileum (88). As a result of all such limitations, FAO recommended digestible indispensable amino acid scores (DIAAS) to replace PDCAAS. In DIAAS method, the digestibility is generally based on the true ileal digestibility in rat or pig models, but most preferably in humans. The pattern of reference to amino-acid scoring of human milk is used for the development of IF (80). Moreover, the source of protein used in IF must also contribute to the required levels of conditionally essential amino acids (61). Mathai et al. (88) reported the values of DIAAS demonstrated in pigs for WPI, WPC, SPI, SMP, and milk protein concentrate (MPC) were 67, 71, 81, 68 and 85, respectively. The drawbacks of this study included the raw protein as a feed, so the study was not reflective of the proteins present in IFs which undergo numerous processing during manufacturing. In another investigation by Maathuis et al. (89), the kinetics of DIAAS and true ileal digestion of protein were determined for human milk (HM), goat milk-based IF (GIF), and cow milk-based IF (CIF). The results showed that the protein quality was not significantly different between HM, GIF and CIF. Still, the protein digestion kinetics of GIF was more comparable to HM than the CIF. The Biological value (BV) of milk is 91%, with the BV of lactalbumin at 104% and casein at 77% (90, 91). In the case of goat and sheep milk protein, BV is 90.9 and 97%, respectively (92, 93). The BV of milk protein can be affected by processing like sterilization due to the loss of lysine and methionine (94). Besides, gamma irradiation also leads to a fall in BV (95).

Protein hydrolysates and peptides generated from major milk proteins, *viz.*, whey and casein proteins, exert immunomodulatory effects involving antibody synthesis, lymphocyte proliferation and cytokine regulation. Immunomodulatory peptides generated from chymosin-pepsin hydrolysis of milk include β-CN f193-202, f191-193, f63-68 (immunopeptides), and α_s1_-CN f194-199 (α_s1_-immunocasokinin) (57). Sharma et al. (96) reported some goat milk immunomodulatory peptides, namely, dipeptidyl peptidase, Interleukin 12 subunit, transcription factor A, oligodendrocyte transcription factor 2. The authors also outlined their biological significance in signaling processes of the cytokines and generating cytotoxic lymphocyte, consecutively enhancing immunity. Interleukin 6 (IL-6) is a cytokine that plays a role in hematopoiesis, inflammation, and immunological regulation. Diseases are associated with greater levels of IL-6 expression. Goat milk peptides are capable of binding IL-6 receptors, owing to immunomodulatory activity of these peptides (97). Donkey milk peptides have also been shown to increase the cytokines involved in the onset of acute local inflammatory response and the regulation of innate immunity. Furthermore, whey protein fraction of donkey milk has a molecular mass greater than 10 kDa and stimulate the productivity of certain immune regulatory cytokines like interferon γ (IFN-γ), interleukin-2 (IL-2) by murine splenocytes (98). Ma et al. (99) regarded β-lactoglobulin_40-60_ from cow milk as an immunomodulatory peptide which directs T lymphocyte activation toward the Th1 phenotype. It can be concluded that there are numerous immunogenic peptides in milk derived from different species. All such components can be isolated and later incorporated while formulating IF based on the varied growing needs of infants.

### 3.3. Fat

The primary energy source in IF is fats, providing nearly half of the infant’s requirement of energy (100). According to the current guidelines of EU legislation, the fat content of IF must range between 4.4 to 6.0 g/ 100 kcal. In addition, the minimum requirements for essential fatty acids like α-linolenic acid and linoleic acid are also set, which the infants cannot synthesize. Hence, their diet must supply these (61). About 70% of bovine milk has saturated fatty acids. The most common fatty acids are the odd chain fatty acids, butyrate and conjugated linoleic acid (58). Arachidonic acid (ARA) and docosahexaenoic acid (DHA) have roles in the plasma membrane constituent’s development and are lower in bovine milk when compared to human milk. Infants fed with ARA and DHA have lower incidences of allergies, nasal congestion, bronchitis, upper respiratory tract infections, cough, diarrhea, eczema and contact dermatitis (101). In human milk, palmitic acid accounts for approximately 10% of the total energy intake of infants. Henceforth, it is a key nutrient for the development of IF (102). Incorporating various structured triglycerides in IF has been associated with reduced intestinal inflammation and colic instances, modified gut microflora, and improved bone development (103). However, more research is still required to determine the exact PUFA ratio and dose for optimal development and growth of cell-mediated and humoral immunity (101, 104).

Hageman et al. (58) compared and reviewed the role of vegetable oil and bovine milk in the nutrition of infants. The researchers suggested that blending vegetable oil and bovine milk could impart additional health benefits associated with various nutrients possessing similar attributes to human milk fat. This developed model utilizes the specific composition of oils and fats (triacylglycerol, polyunsaturated fatty acids and fatty acids). It also determines the degree of differences and similarities to human milk, ultimately leading to the development of realistic substitutes. A study conducted by Mehrotra et al. (105) revealed that the long-chain saturated fats present in the vegetable oil-based IF are associated with the formation of calcium fatty-acids soaps, which in turn contributes to constipation. Their work suggested that reducing palmitic acid may be a solution for improving stool consistency.

In caprine and bovine milk-based IF, the proteins responsible for the stabilization of fat micelles are whey proteins and casein (106). These protein fractions lead to heat coagulation stability, emulsifying ability and emulsifying stability while processing IF (107, 108). The composition and size of the lipid globules of the lipid/water interface have been demonstrated and shown to affect digestibility (109). There have been attempts to modify the lipid/water interface composition, for example, modification in the phospholipid content of IF via adding the milk phospholipids (110). Since the structure and composition of milk fat globules (MFGs) differ between human, bovine and caprine milk. It is therefore necessary to evaluate the MFGs of these milks, in order to explore a more suitable substitute for breast milk. The content of saturated fatty acids is higher in bovine & caprine MFGs, which is about 60% greater than in human MFGs, but unsaturated fatty acids (C18:2) in human MFGs are seven times higher (111). Furthermore, fat globules of donkey milk are very small (average: 1.92 μm). Their small size represents a bigger surface accessible for lipase action, which may help explain its higher digestibility (112). There is considerable variation in the mean size of MFG for human (4.2–5.1 μm), caprine (2.2–3.9 μm), bovine (2.5–5.7 μm) & ovine mature milk (2.8–4.0 μm) (113). In case of caprine-based milk IF, mostly the goat milk is used as a raw material to achieve desired protein content. IF having goat milk also retains components of Milk Fat Globular Membrane (MFGM) and are linked to metabolic, cognitive, and immune development of the infant (114).

Exosomes are another class of secreted biomolecular nanostructures of lipid bilayers with size ranging from 30–150 nm. These extracellular vesicles are linked to human metabolism, physiology and immunomodulation and have remained as an excellent carrier of biomolecules like proteins, lipids, mRNA, DNA etc. (115). Hock et al. (116) reported that milk derived exosomes (MDEs) promote the viability of intestinal epithelial cells, their proliferation and cell activity. The findings also recommended the use of MDEs as a preventative in the treatment of necrotizing enterocolitis, a lethal disorder affecting the intestines of infants. Moreover, higher proportions of sphingomyelin and phosphatidylserine are being reported in bovine and human exosomes as compared to MFGM (117). The camel milk exosomes have anticancer effects possibly via inhibition of oxidative stress and induction of apoptosis, metastasis and angiogenesis in tumor microenvironment (118). Yak-MDEs help in the improvement of GI development under hypoxic conditions (119). Still, the removal of fat globules and cream before long-term storage of milk is necessary to acquire a higher yield of MDE (120).

### 3.4. Minerals and vitamins

The human body cannot synthesize many vitamins and minerals, so these must be supplied in the diet. Both caprine and bovine milk are richer in minerals that can contribute to a high load of renal solute in infants, which leads to a higher risk of hypertonic dehydration (36, 121). Various minerals like phosphorous, calcium, sodium, potassium, magnesium and chloride are added in the form of citrates, chlorides, carbonates, phosphates or hydroxides (33). Reducing the protein content in caprine and bovine milk can also lower the ash content, making it suitable for feeding infants. In some cases where the innate mineral levels derived from carbohydrate sources are insufficient, the fortification of mineral salts is necessary. But it is preferable to amplify the amount of intrinsic minerals because the fortified minerals can lead to instability of IF (36). Besides, there is a reduction in bovine milk heat stability by soluble calcium salt addition. This occurred due to increased ionic Ca and decreased pH levels, which in turn led to reduced heat stability, resulting in the formation of undesirable firm coagulum when sterilized (122). Goat milk contains higher free calcium ions because of insufficient citrate levels. Hence, it is necessary to add phosphate and citrate salts for the enhancement of heat stability while preparing the IF from goat milk (36).

Like minerals, IFs are also fortified with vitamins to meet the requirements of infants. The administration of vitamins is not mandatory, though if infants drink less than 500 mL of IF per day, additional A, D, and C vitamins are recommended (123). While manufacturing IF, the ingredients are exposed to several heat treatments, which causes the deterioration of heat-labile vitamins like thiamine and Vitamin C. Therefore, it is essential to compensate for all such losses while processing by fortification. Heat-labile vitamins must be dry-blended with all powdered ingredients after heat treatment in order to avoid such issues (36, 121). As stated by Wang et al. (124), with the addition of vitamins, metal ions and PUFA, the thiobarbituric acid value increased, and the peroxide value decreased in the early storage periods, indicating oxidative deterioration in IF.

Both vitamins and minerals possess the capacity to elicit a particular immune response known as immunogenicity. This depends upon molecular weight, chemical complexity, route of exposure and dose of immunogen (125). Morante-Palacios et al. (126) demonstrated that vitamin C enhances epigenomic reprogramming of nuclear factor-kappa-light-chain-enhancer of B cells (NF-κB), which in turn boosts immunogenic properties of the dendritic cells. Likewise, vitamin E affects the immune system via modulation of protein kinase C. Vitamin A is also involved in immune system development and plays a regulatory role in humoral immunity and cellular immune regulation (127). Calcium, selenium, Phosphorous, and iodine minerals are essential for brain function development; they also help in strengthening the immune system (128). In addition, the low availability of phosphorus weakens the immune responses. Manganese is another micronutrient whose deficiency in the body may lead to impaired antibody production. Sulfur plays a significant role in transport across membranes, biocatalytic processes and immune functions (129). However, it must be acknowledged that excessive consumption of some minerals can have a negative impact on the immune system (130). Molska et al. (131) concluded that even though the concentration of minerals was higher in IF when compared to human milk yet, their absorption was lower.

Venema et al. (132) demonstrated the importance of lower gastric phase pH for the bioaccessibility and solubility of minerals. Bioaccessibility is the fraction of compound liberated during digestion and made available for absorption. Venema et al. (132) also validated that the type of mineral salt had a direct impact on bioaccessibility; salts with a higher solubility (i.e., dipotassium phosphate, calcium glycerophosphate) result in better bioaccessibility as compared to inadequately soluble salts (i.e., calcium carbonate, calcium phosphate). Besides, on average, the bioaccessibility of Zn and Fe is lower than that of other elements. In contrast, the elements K and Mg are known for their higher bioavailability and efficient utilization (133). To overcome the problem of low bioavailability, complexing mineral with certain organic compounds like amino acid chelators has been exploited. For example, caseinophosphopeptides are widely used in the formulation of mineral enriched IF and can improve the bioavailability of Ca, Zn, and Fe (134). Furthermore, the bioavailability of Fe can be enhanced by fortification of water-soluble Fe compounds in IF, such as ferrous fumarate, ferrous sulfate, ferric pyrophosphate or ferric ammonium sulfate (135).

Although the low bioavailability of carotenoids from IF is unclear but can be due to either micellization or poorer release from the matrix, cellular uptake & transport, or further biodistribution and absorption. Interestingly, the bioaccessibility of carotenoids has been reported not to vary significantly between IF and mother’s milk (136). The bioaccessibility of folic acid and vitamin C from IF is also low compared to breast milk, indicating that vitamin C can be encapsulated in a way that it is not quickly released or react with other ingredients of IF. Also, as far as vitamin A is concerned, the lipid content in the IF influences its bioaccessibility (137). Regarding the more bioavailable source of folate, calcium L-methylfolate is safe and is recommended for use in IF supplementation (138). Microcapsules can be prepared by loading vitamins to improve their bioavailability (135). Otadi and Zabihial (139) formulated a heat-cured vitamin E microcapsule with ethyl cellulose whose release was 10–12% slower than a typical microcapsule. Therefore, all the vitamins and minerals must be evaluated well with their effects on the infant’s gut and their ultimate absorption. Secondly, they must be quantified accurately in infant formula using advance analytical tools like liquid chromatography mass spectrometry (LC–MS) or High Performance Liquid Chromatography (140, 141).

### 3.5. Other ingredients

#### 3.5.1. Lactoferrin

Lactoferrin is one of the most significant bio-activators in milk and other external secretions. It plays a variety of biological tasks, including modulation of the immune responses, iron absorption regulation, antiviral, antimicrobial, antioxidant, anti-inflammatory and anticancer activities (142). Lactoferrin was also successfully added to the formulations of IF, and the resultant formula was safe and tolerated well by the infant, with an expected growth pattern (143). Numerous preclinical investigations using rat and piglet models have shown improved learning and memory capacities due to the lactoferrin-enriched formula (144, 145). According to Wazed et al. (146), the thermal treatment showed better lactoferrin retention than the High-Pressure Processing (HPP) treatment. At a higher temperature, substantial denaturation of lactoferrin was observed. Ultimately the High Temperature Short Time (HTST) pasteurization retained the best quality of Lactoferrin, confirming its possibility to be added to the products of HTST pasteurization.

#### 3.5.2. Osteopontin

Osteopontin is another multifunctional protein involved in many biological processes, including cell proliferation, bone remodeling, immune-modulatory functions and biomineralization. The levels of osteopontin in bovine milk are significantly lower when compared to human milk, and only traces are found in infant formula. This protective mechanism may be an essential factor in a breastfed infant’s ability to ward against sickness. Osteopontin, like lactoferrin, is resistant to *in-vivo* gastric digestion, and this characteristic is maintained across mammalian species (65). Donovan et al. (147) using rhesus monkeys, reported that the consumption of a formula containing bovine osteopontin had an impact on the expression of many regulatory genes. Moreover, this change produced an expression profile resembling breastfed newborns. This research implies that osteopontin might have health advantages. All these findings support the idea of osteopontin fortification in IF. Further, the FDA recommendation for IF supplementation is 160 mg/L osteopontin (148).

#### 3.5.3. Probiotics and prebiotics

Most of the probiotic strains incorporated into IF are isolated from fecal or food microbiota (21). As validated by several studies conducted so far, the *Lactobacillus* and *Bifidobacterium* strains are considered to be the most suitable probiotics for infants. Furthermore, *Streptococcus* and *Propionibacterium* are other promising strains to be used as probiotics (149). Prebiotics are indigestible OSs that stimulate bacterial growth and function (150, 151). The infection and stool rate are also lessened in the case of infants fed with prebiotic-supplemented IF (152). It was also found that infants fed with HMO-supplemented formula had a decreased incidence of bronchitis and use of medications such as antibiotics and antipyretics (153). Nowadays, there are seven approved food-grade OSs for inclusion in IF involving lactulose (LOS), inulin, polydextrose (PDX), 2′fucosyllactose (2′-FL), galacto-OS (GOS), fructo-OS (FOS) and N-neo-tetraose (LNnT) (154). Nevertheless, IFs are generally fortified with FOS, GOS and polydextrose prebiotics (151). The addition of lacto-N-neo-tetraose and 2′fucosyllactose has contributed to narrowing the compositional gap between IF and human milk (155).

#### 3.5.4. Choline

Choline is the precursor of the phospholipids, acetylcholine and platelet-activating factor (156). In the case of lactating women intake of 550 mg per day of choline is required, whereas 450 mg per day is needed for pregnant women (157). Choline presence alters the spinal cord and brain structure and can lower the risk of defects in neural tubes (158). Choline supplementation during pregnancy, at nearly twice the recommended dose (930 mg), speeds up the infant’s ability to process information (159). Additional choline may enhance cognitive, affective and neurological functioning when consumed by mothers carrying Down syndrome fetuses (160). Inadequate choline levels can hinder vitamin B12 and folic acid metabolism (65). The IF must contain 25 to 50 mg per 100 kcal of choline, as recommended by the EU legislation (61). If the raw materials lack choline, the IF is fortified with choline salts like choline chloride (80).

#### 3.5.5. miRNA

MicroRNA (miRNA) are short non-coding molecules involved in post-transcriptional gene regulation and have been discovered in cells, lipid fractions and skim milk of humans, which originate from mammary glands (161, 162). miRNA is involved in cell proliferation, apoptosis, differentiation and immune response (163, 164). These are bioavailable in human milk and are transmitted to infants during lactation (165). miRNA is enriched in bovine and human milk but is present in a lesser amount in caprine milk (166). Much research has been conducted to highlight the effects of miRNA on obesity, diabetes, inflammation and cardiovascular diseases in the offspring tissue (167). In addition, Yun et al. (168) confirmed the presence of immune-related miRNAs in both colostrum & mature milk of human, bovine, as well as caprines. Yet, no miRNA has been detected in human or bovine milk-based IF to date. This might be due to the degradation caused by heating or homogenization (169). Although according to Golan-Gerstl et al. (164), pasteurization has a minor effect on the miRNA 148-3p profile expression in skim milk and fat fractions of caprine and bovine milks.

#### 3.5.6. L-carnitine

IF must also contain a compound called L-carnitine, which is an essential nutrient for neonates as they cannot synthesize it for a short period of time. It is a water-soluble molecule that resembles vitamins and is present in various plants, microorganisms and mammalian species. It plays a primary physiological role in the metabolism of fatty acids (80, 170). According to EU legislation, the minimum concentration of 1.2 mg/100 kcal L-carnitine must be present in IF (61). Mikhael (171) suggested carnitine is an essential ingredient of IF, and its deficiency can cause anomalies or infections in infants.

#### 3.5.7. Lutein

Lutein is a carotenoid present in human milk. Its concentration varies according to the maternal diet, i.e., with the intake of vegetables and fruits (172). The function of lutein is to be a structural component of the eye, to act as a filter for blue light and as an antioxidant. It plays physiological and biological roles in an infant’s visual development and function (172, 173). More than two-thirds of the carotenoids, specifically lutein, are enriched in varied sections of the human brain, i.e., temporal and occipital, frontal and cerebellum cortices (174). The bioavailability of lutein in human milk is almost four times higher compared to lutein-fortified IF (175). Recent studies have stated that lutein supplementation in IF is safe and supports cognitive and visual development (176, 177). In present days there are several infant formulas containing lutein (178). Among all the available lutein, crystalline lutein-zeaxanthin is the most popular commercially available form of lutein (179).

#### 3.5.8. Taurine

Another nutrient, namely taurine, is sulfur-containing amino acid, possessing a sulphonic-acid group and is manufactured from either monoethanolamine or ethylene oxide. It is a beneficial dietary supplement commonly added to nutritional supplements, infant formula, energy drinks and pet foods (180). Taurine is present in higher amounts in human milk than in bovine milk; therefore, the IFs are usually fortified with it, with levels not exceeding 12 mg/100 kcal (61, 121). It is believed to play a role in intestinal fat absorption, hepatic function and the development of the long-term nervous system. Long-term administration of formula milk lacking in parenteral nutrition or taurine has been linked to hepatic cholestasis, retinal degeneration, lower fat absorption, decreased bile production and delayed auditory maturation (181).

#### 3.5.9. Ganglioside

Gangliosides are commonly found in the cell’s lipid membrane and are sialylated glycosphingolipids. They play a role in gut integrity, neurological development, immune cell signaling, preventing infections and intracellular trafficking (182, 183). The supplementation of disialoganglioside has shown a favorable effect on the infant’s neurological development. Infants with an age of 2 to 8 weeks, when supplemented with 2–3 mg ganglioside per 100 g of IF, showed increased General IQ, Performance IQ, and Hand & Eye Coordination IQ on Griffiths scales (184). Moreover, very few studies have been directed until now to understand this bioactive compound’s tolerability, efficacy and safety (101).

#### 3.5.10. Inositol

Inositol is a carbocyclic sugar alcohol and is reported to play a role in many biological functions like phospholipid production, cell osmolarity regulation and cell signaling (185). It is naturally made in humans from glucose and is only half as sweet as sucrose (186). Human milk contains more inositol than bovine and caprine milk (187). Following EU legislation, the IF must contain a minimum of 4 mg per 100 kcal of inositol (61). Inositol is helpful in treating preterm infants who have infant respiratory distress syndrome or are at risk for it (188). Myo-inositol also prevents neural tube abnormalities, especially when paired with folic acid (189).

## 4. Manufacturing of infant formula

**Figure 2:**
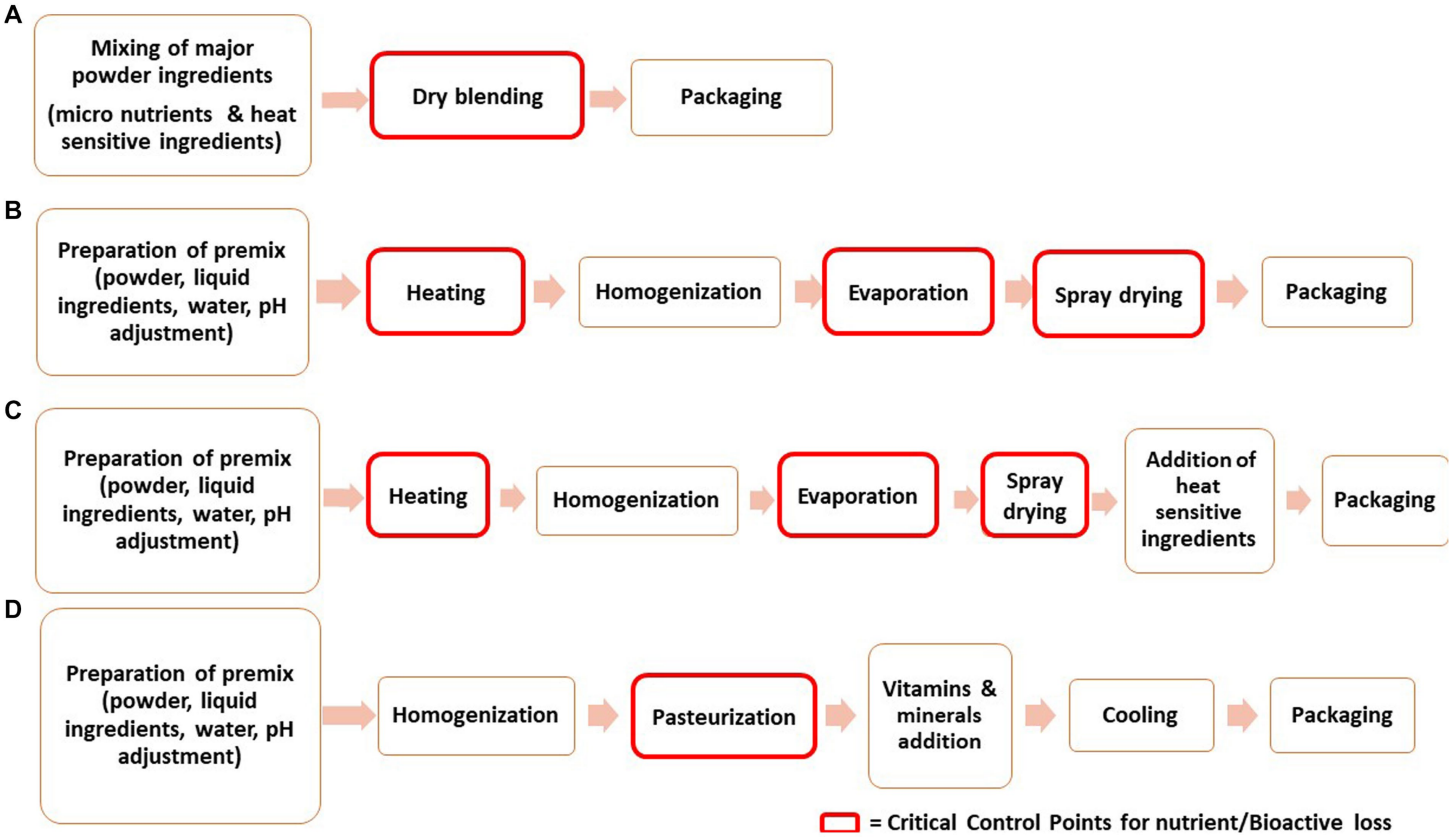
Schematic diagram presenting the processing steps involved in manufacturing of Infant Formula **(A)** Dry-blending, **(B)** Wet-blending, **(C)** Combination of dry and wet blending **(D)** Liquid infant formula.

Manufacturing of IF involves multifarious processing techniques resulting in different forms of the formulas. The most commonly available form in the market is the powder one. The liquid formula has certain drawbacks like lower shelf life, demands extra packaging care to avoid contamination and has elevated prices (212). For the production of powdered IF, diverse technologies are employed. Generally, the powder IF is manufactured by dry blending and wet blending (213, 214). On an industrial scale, the combination of both dry and wet blending is implemented (33). Liquid infant formula is also manufactured by time temperature processing, majorly by ultra-high Temperature for extending shelf life (215). The common processing steps involved in the manufacturing of infant formula are schematically presented in Figure 2.

### 4.1. Thermal processing

Traditional thermal methods have been an important tool for the processing by application of heat to acquire powder products or potential pasteurized and sterilized effects. It is unambiguous that by this treatment, there is an efficient reduction of microorganisms resulting in a shelf-stable product (216). Moreover, it is also important to keep the proper balance between heat treatment and control of chemical changes (217). These are based on heat generation outside the product and later heat transfer by either convection or conduction mechanism. The heat application could be by direct or indirect contact with the matrix. On top of that, in thermal processing, an absolute knowledge of thermophysical and thermal properties is vital to determine product behavior. These characteristics are heat diffusivity, penetration coefficient, thermal conductivity, chemical composition, dielectric constant, and modulating product structure. Moreover, these treatments are cost-effective, and numerous studies have been conducted so far to optimize a particular temperature and time combination treatment (218).

#### 4.1.1. Dry blending

In “dry blending,” all the dry ingredients are first mixed well and then packed (219). A large-scale blender or Ribbon blender is employed for the uniform blending of large batches. The blend is then passed through a sifter to remove extraneous matter or oversized particles. The blended material passes to the packaging line and is filled into their respective packaging material by a filler hopper (217). The empty flexible pouches are flushed with nitrogen to prevent oxidation (220). This method has some advantages, like lower cost of equipment and maintenance. Further, there is less energy expenditure during production (221). Besides these benefits, microbial contamination is possible as it depends on raw material quality for assembling the final product. Moreover, considerable problems are related to post-processing contamination with *Enterobacteriaceae* and *Salmonella* (222, 223). As the densities of ingredients used vary, they tend to segregate during storage and transportation, leading to a nonhomogeneous state which is not preferred by the consumer. Secondly, the solubility and wettability of dry-blended IFs are not as good as wet-blended IF (217).

#### 4.1.2. Wet blending

To minimize the microbial risk associated with dry mixing, the wet blending method offers better process control and monitoring in manufacturing steps (213, 214, 224). Due to better control, wet blending produces powdered IF with better microbial and physicochemical properties (225). In wet mixing, the dried ingredients are dissolved in skimmed milk or preheated water. Typically, a low Total Solid level (20–30%) wet-mix is obtained to prevent problems related to high viscosities (224). Subsequently, the mix is pasteurized and then homogenized. The pasteurization of the mix is carried out at 75°C to 100°C for 18 s (226). The effect of heating after and before homogenization was evaluated by Buggy et al. (227) and the results depicted that the particle size of aggregates/droplets was smaller in the case of heating application before the homogenization step. Thus, pasteurization is carried out before homogenization. The homogenization process is generally implemented to reduce fat particle size, increase surface area and produce stable emulsions (64). But it also includes stabilizing the protein layer (228). The wet mixes are subjected to two-stage homogenization, applying 13.8 MPa and 3.5 MPa pressure in the first and seconds stage, respectively (64, 84, 213).

Afterward, this preparation is concentrated in a vacuum evaporator to achieve 45–55% total solids and then spray dried (223, 224). The main aim of the evaporation step is to reduce the spray drying cost (224). Additionally, the physicochemical properties of IF are also better. In the dairy and food industries, falling film evaporators are frequently used for evaporation. IF wet-mix is typically concentrated at 50°C and 70°C (64, 213). The spray drying is characterized by the atomization of concentrated premix into tiny droplets of size 10–400 μm. The water molecules of the liquid are evaporated by heated air flow with inlet and outlet temperatures ranging from 180–200°C and 80–100°C (229). As this exposure is very short, so the core temperature does not exceed 45°C. In order to maximize the thermal efficiency and evaporative capacity of the dryer, it is crucial to choose the proper inlet–outlet temperatures (230, 231). Inlet–outlet temperatures also affect water activity, moisture content, glass transition temperature and particle size (229).

In the last phase, the dried powder is recovered in the cyclone separator (232). The recovery system is coupled with adequate packaging steps resulting in minimum microbial contamination. The appropriate packaging method extends the product’s shelf life by preventing oxidation, deterioration and particle agglomeration (212). The resulting IF is sensitive to browning, lipid oxidation and caking induced due to moisture. Thus, commonly used packaging materials are polyolefin-coated metal cans (233). An and co-workers (234) demonstrated the effect of the modified atmosphere on the quality of IF packed in metal cans. The results showed that a high CO_2_ environment helped in oxidation prevention and increased the survival of incorporated *Bifidobacterium*. Although MAP packaging is the most widely used technology for powdered products, it cannot generally attain complete oxygen removal (235). To strengthen MAP, the incorporation of antioxidants in the polymer can be effective for inhibition of oxidation and scavenging of headspace oxygen (236). Jo et al. (237) incorporated ascorbic acid in Al- laminated film pouches with CO_2_ flushing under MAP conditions and then compared it to plain film. This resulted in further lipid oxidation suppression and extended the storage period of powdered IF. Hence, newer and more advanced packaging technologies are required to extend the shelf life and keeping quality of IF.

#### 4.1.3. Processing of liquid infant formula

For the manufacturing of liquid IF, the initial processing steps involved are similar to wet blending (212). At first, the emulsion is formed by mixing vegetable oil with 90°C preheated water and then lactose, milk, OSs, WPC & fat-soluble vitamins are added. Homogenization is done for the stabilization of the emulsion. After this step, emulsion is pasteurized (72°C for 10 s) or sterilized (12°C for 3 s), followed by rapid cooling at 10°C (215, 238). A careful selection of thermal treatment is necessary to optimize the physiological effects possessed by bioactive peptides, which are released after the digestion of IF (239). All minerals and water-soluble vitamins are added to the obtained emulsion. Eventually, this solution is diluted with water to achieve the desired nutrient concentration of the liquid formula. Later, for even mixing, homogenization is again carried out at 240 bar, followed by a sterilization step at 136°C for 35 s (238). The sterilized formulation is cooled to 25°C and aseptically filled in PET (polyethylene terephthalate) bottles (215).

### 4.2. Challenges associated with thermal processing for manufacturing of infant formula

Processes like pasteurization, homogenization and spray drying have the most pronounced impact on the properties of infant formula due to the interactions between protein-carbohydrate, protein–protein and protein-lipid. These thermal processes may also be responsible for the interaction among other components and the changes in the physicochemical, structural and reconstitution properties of IF (240). Subjecting the formula to several heat processes while its production promotes the Maillard reaction and aggregation or denaturation of proteins, as they are most sensitive to heat (241). In particular, when aggregation occurs, there is a rise in viscosity, a decrease in emulsion stability and a reduction in the overall performance during processing (242). Most proteins lose their biological activity due to aggregation, which ultimately encourages protein coagulation (243–245). Casein protein is more heat stable when compared to whey (246). Nevertheless, intense heating of casein can lead to its cleavage, causing dephosphorylation. Additionally, there is a loss of the protective function of κ-casein if glycosylated. Further, as β-casein is mainly responsible for the bioavailability of zinc and calcium, its functionality loss may result in many future problems in infants (247).

Whey proteins are more prone to heat treatment, which could change their nutritional and functional properties (248). Milk protein solubility decreases when there is an interaction between whey protein and casein micelles leading to their aggregation or dissociation. This ultimately results in the alteration of functionality and hydrophobicity of reconstituted products (243–245). The most sensitive proteins are Igs, and these are present in lower concentrations (225). Upon heating, the bioavailability of zinc and calcium declines as the α-lactalbumin loses its ability to bind with these minerals (249). Although there is no presence of β-lactoglobulin in human milk, still when cow’s milk-derived IF is exposed to heat, it can become insoluble or can get denatured (250). To prevent the aggregation of β-lactoglobulin during thermal processing, the increment of α-lactoglobulin concentration can be an alternative (227). Protein denaturation induced by heat treatment can modulate protein allergenicity. This can occur due to exposure or masking of epitomes, depending on heat treatment intensities (248).

The Maillard reaction can take place when IF is heat treated as it contains proteins and sugar. It is a chemical reaction between the reducing sugar’s carbonyl group and free amino acids, forming Schiff’s base, i.e., lactosyl-lysine (231). Lysine is the most reactive amino acid, but sugars can also react with histidine, arginine, tryptophan and methionine (251, 252). The Schiff’s bases formed are chemically unstable and are susceptible to additional isomerization, known as Amadori rearrangement, which results in the creation of lactulose-lysine (the Amadori product). Consequently, the bioavailability of lysine can be affected, causing nutritive loss (81, 251, 253). Prolonged heating in acidic or neutral conditions, there is a formation of various furfural compounds like 5-methyl-2-furaldehyde, 5-hydroxymethylfurfural, 2-furyl-methyl ketone and 2-furaldehyde (254, 255). N^ε^-carboxymethyl-lysine is an advanced glycation product that can cause potential pro-oxidant and pro-inflammatory health effects (256). Aldehydes, reductones, furfurals and some other intermediate products tend to react with amines and form dark polymeric compounds of higher molecular mass called melanoidins (257). Melanoidins have the ability to chelate metals, which can be harmful if they influence nutritionally essential metals like Zn, Ca, Cu, Mg, and Fe and thus impair mineral absorption and metabolism (258, 259). Over time, there has been a revolution in newer techniques for studying the generation of all these undesirable compounds formed during thermal processing. For example, the advanced glycation end-products (AGE) were predicted by the Molecular Transformer model curated with data from the literature (260).

MFGM has recently been permitted to be incorporated into the IF. Heat treatment can also affect it, leading to its enhanced permeability and hence reducing its stability (261). A combination of thermal and homogenization treatments causes fat structure differences in terms of size, interfacial architecture, composition and fatty acid profile (262, 263). Also, there is phospholipid breakdown in heat treatment with a consequent increment in inorganic phosphate (264). Heat processing results in the loss of vitamins and minerals and the isomerization of lactose to lactulose (143, 265, 266). Heat treatment intensities determine the extent of alterations in the mineral balance between serum and colloidal phases (246). Compared to macro-elements, significantly less information is available on heat treatment effects in trace mineral concentration and distribution. Nonetheless, trace minerals can also be lost during heat treatment or in other unit processing involving heat, such as in fouling deposits during drying and evaporation (267, 268). Moreover, due to heat, there is partial degradation of water-soluble vitamins like C, B_1_, B_6_, B_12_, some hormones and unsaturated fatty acids. Both drying and evaporation operations can be detrimental to oxygen-sensitive components, including most fat- and water-soluble vitamins and unsaturated fatty acids (264).

### 4.3. Non-thermal processing of infant formula

With the aim of combating organoleptic and nutritional changes occurring during heat processing, various nonthermal technologies have been introduced in the manufacturing of infant formula. These novel techniques include HPP, Ultrasound, Ionizing radiations, Pulsed electric field (PEF), Ultraviolet irradiations and Cold plasma (269). Over the past few years, numerous researchers have been actively working on non-thermal technologies to study their impact on pathogenic microbes and nutritional profiles. Yet, the aforementioned technologies have a limited impact, higher equipment & processing cost, and stringent operating requirements (270). Table 3 summarizes the application of non-thermal technologies for processing Infant Formula and inactivation of microbiological contamination, while retaining nutritional and other quality attributes.

**Table 3:** Application of non-thermal technologies for processing of infant formula and its effects on pathogenic microbes and quality attributes.

Technique	Medium	Treatment regime	Microbes	Key findings	Impact on quality attributes	References
HPP	Reconstituted IF	The pressure: 600 MPa at 4°C for 5 min in combination with chitosan & trans cinnamaldehyde (TC) (@ 1% & 0.05%).	*Cronobacter sakazakii*	Reduction of *C. sakazakii* to an undetectable level. Structural deformations in the interior & cell wall. Release of the intracellular content.	Sensory attributes were similar to control, supporting the feasibility of HPP.	(301)
HPP	Ready to feed IF	Treated with 300, 400, 500 & 600 MPa for time period of 10&20, 10&20, 2&10 and 1&5 min. at 40°C.	–	The highest ratio of α- lactalbumin to β- lactoglobulin (β -Lg) was achieved after HPP (600 MPa for 5 min at 40°C)	Overall, a significant decrease in β -Lg was caused by the interaction of HPP and heat.	(302)
HPP	Reconstituted IF	Treated with 600 MPa pressure at 4°C for 5 min. Addition of 0.1% TC.	*Bacillus cereus*	When combined with TC, HPP demonstrated the highest inactivation rate, confirming the synergistic effect. Damage, deformation in vegetative cells & spores.	A significant difference was observed in flavor because of the cinnamon-like taste.	(303)
γ- irradiation	Powdered & liquid IF	The source used was Co^60^ & carried at −20°C, 4°C with a dose range from 0–10 kGy.	*S. typhimurium*, *L. monocytogenes*, *S. aureus*, *B. cereus* & *E. coli*	Except for *S. typhimurium* & *L. monocytogenes*, all pathogens were very sensitive to irradiation	No impact on chemical composition. Significant increase in lipid oxidation of powder in a dose-dependent manner.	(284)
γ- irradiation	Powdered and frozen infant formula	The source used was Co^60^ with a dose range of 0–10 kGy in combination with 0.5% sodium carbonate, 2.5% sodium citrate, 0.75% citric acid	*S. typhimurium*, *B. cereus*, *E. coli*, *L. monocytogenes* & *S. aureus*,	Alone, gamma radiation was more effective in the frozen formula. Additive-induced radio sensitization of microbes; highest effect by sodium carbonate.	The use of γ-irradiation in combination with GRAS food additives can be a good way to reduce the irradiation time.	(285)
Electron beam irradiation	Dehydrated IF	A dose range of 0–25 kGy one-sided electron beam with energy at 10 MeV at 4°C temperature was applied	*C. sakazakii*	Non-thermal inactivation of *C. sakazakii,* which is a major foodborne pathogen of concern in IF.	Proximate profile remained comparable. No size degradation due to higher dose. Lipid oxidation with 25 kGy dose.	(280)
Electron beam and UV-Pulsed light (PL)	Powdered IF	e-beam dose of 1.5, 5, 10 and 25 kGy with energy at 10,12 MeV. For UV-PL at doses of 4.32, 10.8, 12.98 μJ/cm^2^. Generation of UV-C by xenon lamp.	*C. sakazakii*, *L. monocytogenes*, *S. enteritidis*, *E. coli*, *S. aureus* & *B. cereus*.	10 kGy application of e-beam sterilized the IF. However, PL was not well suited for sterilization because of turbidity issues.	At 10 kGy no significant differences in moisture, total carbohydrates, total fat, protein, ash, and vitamin C content.	(287)
UV-C and HILP	Dried IF	UV unit comprised mercury lamp emitting continuous 253.7 nm UV-C. HILP unit consisted of a Xenon flash-lamp generating pulses of 360 μs at 3 Hz frequency.	*C. sakazakii*, *S. typhimurium*, & *L.* *monocytogenes*	*L. monocytogenes* was most sensitive. HILP inactivated nearly all the vegetative cells. For *B. subtilis* similar result by doubling the fluence. *C. sakazakii* required the highest UV-C fluence.	Retention of sensory and nutritional properties without formation of any toxic residues.	(300)
SC-CO_2_	Dehydrated powdered IF	CO_2_ (99.5%) was injected at 31.1°C temp. & 7.38 MPa pressure.	*C. sakazakii*	The inactivation of pathogens by SC-CO_2_ was enhanced as T & P conditions increased.	There was no significant change in water activity, pH, and color.	(299)
CAP	Non-fat dairy milk powder	Samples were treated for 0 to 120 s, and N_2_ was used as background gas with a power of 480 W.	*C. sakazakii*	The inactivation increased as the flow rate raised. Combination of fluidized reaction system & CAP may effectively combat microbes.	Even after 120 s-CAP treatment, no significant changes in amino acid, color, crystallinity & phenolic content.	(304)
CAP	Powdered IF	He-O_2_ gases used for generating CAP at 474, 659 and 900 W for 2.15, 10, 20 and 40 min	*B. cereus*, *C. sakazakii*	Reduction of spores. When integrated with microwave & heat, resulted in 90% spore reduction.	The potential of CAP on microbiological safety.	(305)
LED	Powdered IF	405 nm LED with an irradiance of 18.94 ± 0.05 mW/cm^2^.	*C. sakazakii*	Decreased pathogen resistance when combined with heat treatment.	LED as a supplementary tool in the preservation of IF.	(306)

CAP, Cold atmospheric plasma; e-beam, Electron beam; HILP, High-intensity light pulses; HPP, High-Pressure Pasteurization; IF, Infant formula; LED, Light-emitting diode; PL, Pulsed light; SC-CO_2_, Super-critical carbon dioxide; TC, trans cinnamaldehyde; UV, Ultraviolet.

The High-Pressure Processing technology finds its potential application in wet mixtures, which are later subjected to spray drying or in the production of non-thermally pasteurized liquid IF (271). Due to economic and technical constraints, the pressure limit is about 600 MPa (272). It can also be combined with other non-thermal technologies to enhance the effect of microbial inactivation (273). As previously said, pasteurization is characterized as a heat-based intervention, and HPP is now included in the definition of pasteurization as a non-thermal pasteurization technology (274). Apart from HPP, PEF is another non-thermal technology applied for IF processing and is generally employed on non-liquid food items (275). PEF effectively eliminates enzymes linked to quality degradation, pathogenic bacteria, and spoilage-causing microbes without diminishing consumer demand (276). The inactivation induced is due to the dielectric breakdown of the cell membrane and electroporation. Several factors affecting PEF are pulse intensity, number of pulses, flow rate, shape and pulse width. Besides these physiological parameters of microbes and conductivity, the temperature can also affect the microbial inactivation rate (277). PEF implies the application of strong electric fields in short pulses in intestines ranging from 10–80 KV/cm for microseconds (278).

The ionizing radiation methods, such as gamma and beta radiation, effectively inactivate foodborne bacterial pathogens while maintaining the macronutrient composition. But, the preservation of the nutritional matrix depends upon the food composition, environmental conditions and irradiation dosage (279, 280). Both the γ and β radiations comprise the kinetic energy of photons and electrons. When it comes in contact with the target, excitation and ionization occur in the form of a physiochemical effect (281). These technologies affect living cells through the induction of physiological, genomic, morphological and biochemical changes (282). One of the significant effects is damage in macromolecules via direct energy dissipation leading to the breakage of double and single bonds, ultimately causing cell apoptosis. On the other hand, the indirect effect involves the formation of free radicals and highly reactive oxygen species, causing damage to cellular material and nucleic acids (283). Gamma irradiation technology has also been applied to infant formula, whether in liquid or powdered formulation, for the inactivation of these pathogens with a dosage range of 0–10 kGy (284, 285). The most commonly used radioactive source are cobalt-60 and cesium-137. On the industrial scale, Co^60^ is mainly utilized for radiation generation from Co^59^, which is a non-radioactive stable metal (286). For Electron Beam, a high voltage power supply is the source of electrons applied to IF with a dose range of 0–25 kGy and 10 MeV energy (280, 287). In order to monitor radiation levels and create regulations on radiation safety, it is crucial to determine the radioactivity levels in IF and the associated doses (288).

Cold Atmospheric Plasma (CAP), or simply the Cold Plasma, among emerging non-thermal technologies, is utilized for sterilization and microbial decontamination. The plasma species are responsible for the oxidation of macromolecules (proteins and lipids) and the destruction of the cell membrane (289). Certain benefits aided by cold plasma consist of lower operating temperatures, minimal destruction of flavor and nutrients and no toxic by-product generation (290). Nevertheless, some studies have shown that CAP impacts the functional and structural properties of various food components (291–294). Gao et al. (295) reported that CP treatment at 70 kV for about 180 s trigger lipids oxidation during storage. Pulsed light also has the potential for rapid inactivation of microbes on food surfaces by application of short pulses rich in UV light (100-1,100 nm). There is no deleterious effect of pulsed light on lipid oxidation and protein composition. Despite microbial inactivation, its drawback is the agglomeration of powder particles due to inter-particle forces developed by moisture absorption. All such parameters must be kept in mind while selecting particular non-thermal processing (287).

Within the infant formula manufacturing sector, bacteriophage application might be seen as a new-generation approach to non-thermal biocontrol. Bacteriophages (phages) are obligatory parasites that lyse live bacterial hosts. The lytic protein produced by the phages can hinder the growth of *Cronobacter sakazakii* and *Staphylococcus aureus* (296). Kim et al. (297) delineated that bacteriophages can control the *C. sakazakii* growth and its biofilm-forming ability when IF milk is treated with two representative bacteriophages (PBES19 & PBES04). The results depicted that bacteriophages can be effectively used to increase food safety in commercial facilities. However, the methods in optimization for enhancing viable phage propagation are not cheap and also require considerable investment when it comes to upscale (65). On top of that, natural antimicrobials such as copper sulphate, lactic acid, nisin, lactoferrin, trans-cinnamaldehyde, caprylic acid, etc., can be considered efficacious in controlling the microbial spoilage of IF (271).

**Figure 3:**
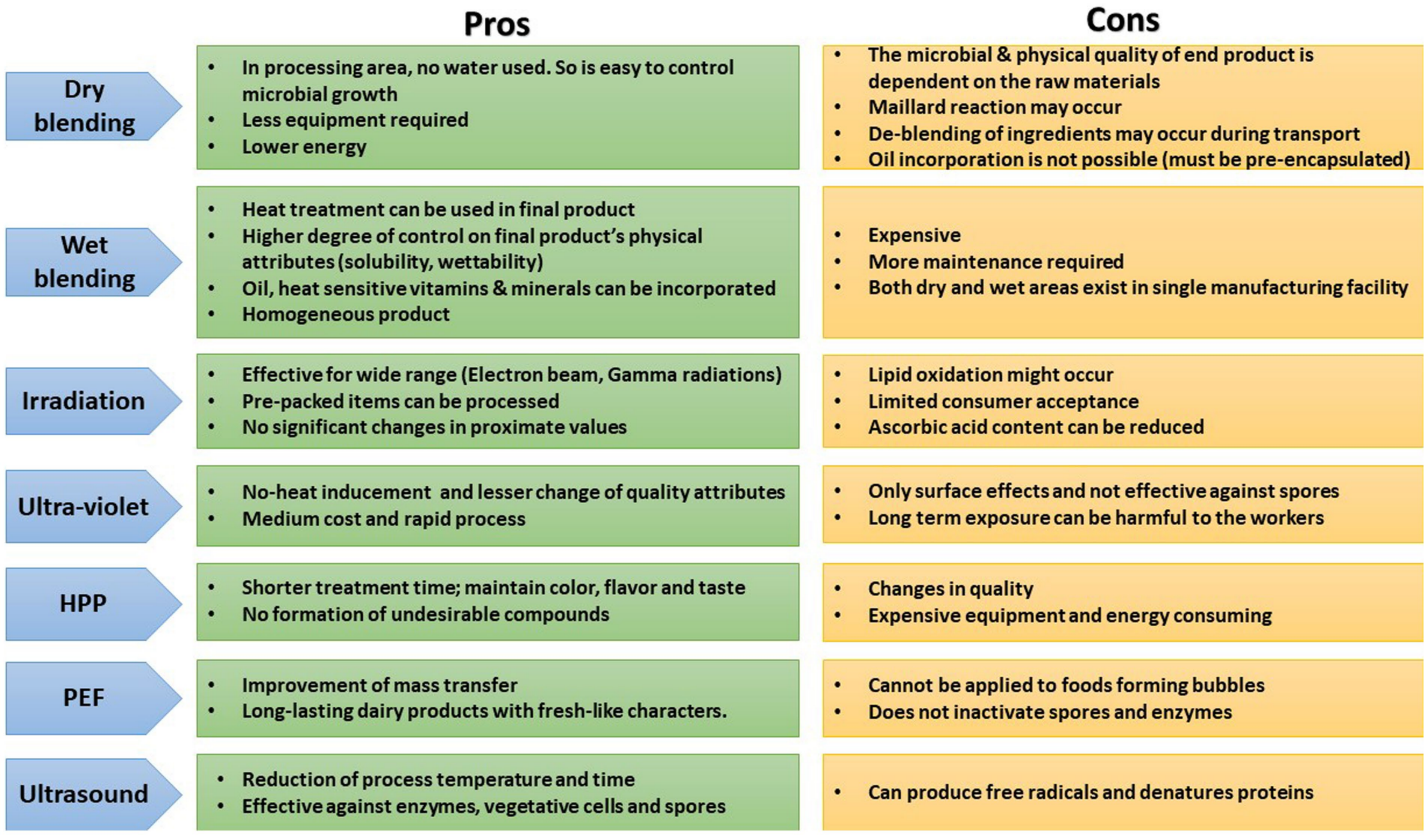
Pros and cons of different processing technologies applied for manufacturing infant formula. HPP, High pressure processing; PEF, Pulsed electric filed.

The use of supercritical carbon dioxide has been an alternative pasteurization technology for many food items. The treatment involves contact of a pasteurized item with super or sub-critical CO_2_ for a defined amount of time in a continuous, batch or semi-batch manner. Supercritical CO_2_ is the CO_2_ at pressure and temperature above its critical point and exists as a single-phase, having the ability to solubilize materials and diffuse through the solids. It is majorly used for the pasteurization of liquid items involving ready-to-feed formulas (298, 299). Further, newer non-thermal technologies based on light, such as broad-spectrum pulsed light (200–1,100 nm) and UV-C (254 nm), are some potential hurdles that can be applied as post-production processes in dried IF. Nonetheless, UV-C is traditionally used for the decontamination of drinking water, wastewater, air and solid foods surfaces. On the contrary, High-Intensity Pulsed Light (HIPL) is a modified version of UV-C and is claimed to be an improved method for UV-C delivery and be applied to powdered IF (300). The advantages and disadvantages of several processing technologies used for manufacturing IF are presented in Figure 3.

## 5. The action of infant formula on infant’s gut microbiota and their possible mechanistic connection

### 5.1. Establishment of infant’s gut microbiome

Infants’ physiological development includes the growth of their gut flora, which should be considered in clinical practice for manufacturing infant formulas (307). The gut microbiota exhibits several functions like the synthesis of vitamins and *de novo* synthesis of amino acids, facilitating the utilization of nutrients, metabolic system’s programming and immune system development etc. It also modulates body composition and infant growth. All of these factors have implications for infants’ short- and long-term health (308). Many studies have reported varied qualitative bacterial flora composition among breastfed infants. In addition, an increase of fermentative bacteria and a decrease in the growth of putrefactive flora was also observed (309). This switch aids in the improvement of intestinal function, such as the absorptive and digestive functions of several nutrients, especially vitamins. Ultimately, there is stimulation of the GI-associated immune system, which reduces the risk of allergies in infants (310).

In the early weeks of life, there are majorly obligatory and facultative anaerobes in the infant’s gut. The establishment of obligatory anaerobes is made possible by the initial colonization of the gut by facultative anaerobes. Later, the microbiota of the gut achieves structural complexity (311, 312). Hence, initial microbial colonization is critical in defining the bacterial flora of adults. Once the microbiota of adults is established, it remains constant, with the exception of potential alterations brought on by a variety of causes, such as a change in dietary habits or the progression of diseases (313). Many longitudinal and cross-sectional studies indicate that undernutrition in childhood is associated with immaturity of gut microbiota, altered diversity, depletion in obligate anaerobes, and enrichment in potentially inflammatory and pathogenic species accompanied by low nutrient utilization (314).

**Figure 4:**
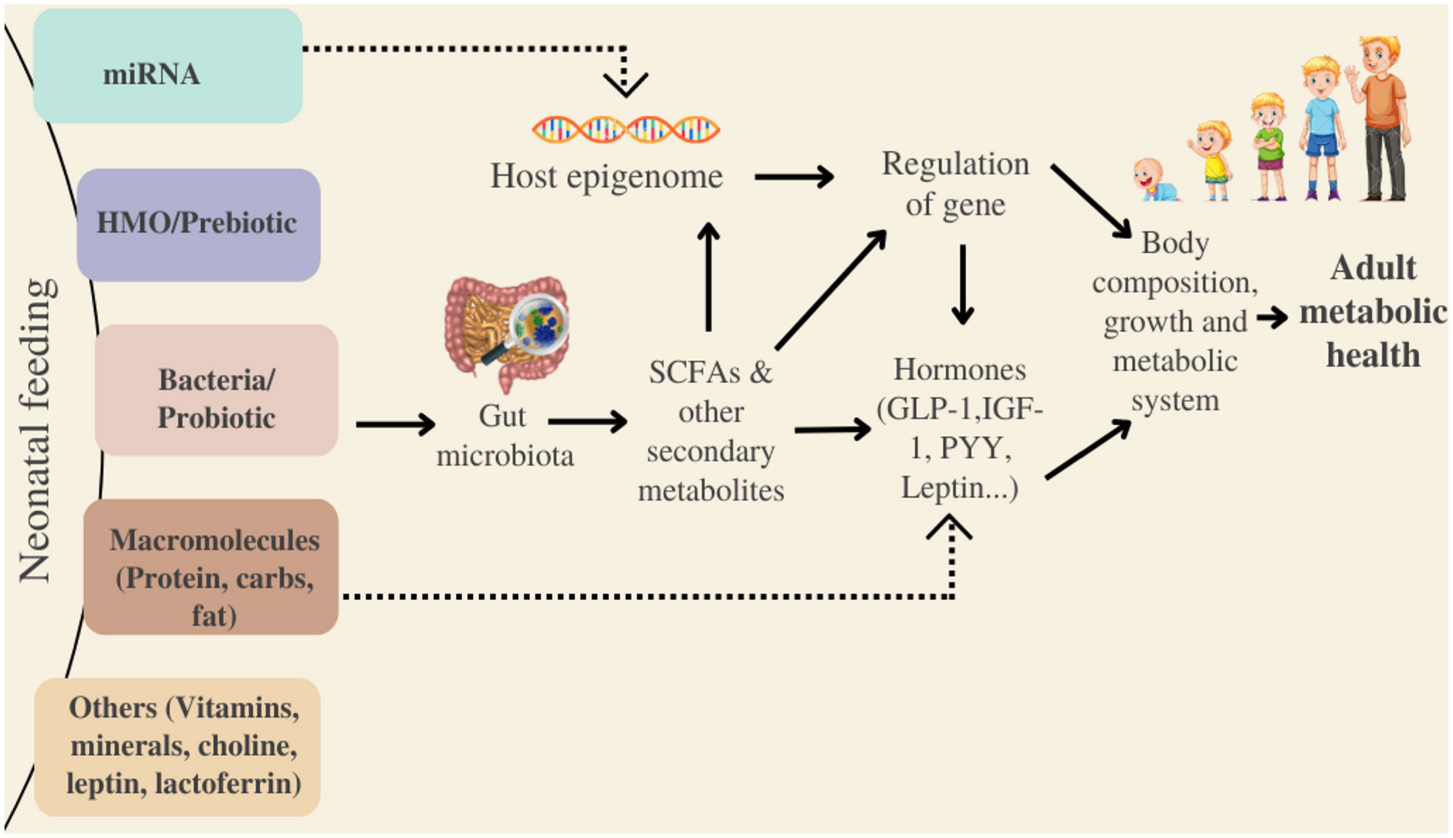
Potential pathway entailing gut microbiota and its link to long-metabolic health. HMO, Human milk oligosaccharides; SCFAs, Short-chain fatty acids; PYY, Peptide YY; IGF-1, Insulin-like factor-1; miRNA, non-coding microRNA; GLP-1, Glucagon-like peptide-1.

Besides, the gut microbiota is also involved in the nutrient-sensing mechanisms of the intestine (315). One of the probable mechanisms underlying the interaction between host metabolism and microbiota is via appetite-regulating hormones (involving ghrelin, leptin, and glucagon-like peptide-1). Gut microbes can produce numerous neuroactive compounds (acetylcholine, dopamine, serotonin & GABA), which can act on both the central nervous and enteric systems. They have the potential to modulate cognitive function and behavior; bind to receptors expressed in the brain (bile acids & indoles). Meanwhile, dietary precursors in the synthesis of host neurotransmitters, like tyrosine (for dopamine) and tryptophan (for serotonin), can be metabolized by microbial enzymes (316). Short Chain Fatty Acids (SCFAs), the end product formed after bacterial fermentation of non-digestible carbohydrates, might be able to alter metabolism and energy harvest through adipogenesis, enteroendocrine cells and production of insulin-like growth factor-1 (317). Host microbial-genomic interaction has recently been found to be an important mechanism associated with copious aspects of human health and diseases. According to metagenomic research, host genes play a role in shaping the microbiome, which in turn, regulates the gene expression of the host (314). The integrative approach by Charton et al. (318) underlined specific bacteria (*Veillonellaceae*, *Prevotellaceae*, *Enterobacteriaceae*, *Rikenellaceae* and *Lachnospiraceae*) associated with commutation of the gut-brain axis. A potential pathway depicting the relationship between neonatal feeding, gut microbiota and the metabolism of the infant is presented in Figure 4.

In addition, it is also necessary to investigate the functionality of gut microbiota belonging to various taxonomic groups and clearer picture of microbiome functionality can be derived from metatranscriptomics (319). A minor overall functional difference, formula-fed infants showed a faster functional maturity when compared to breastfed infants at four months (320, 321). Further, a meta-analysis study by Ho et al. (322) inferred that infants who were not exclusively breastfed had lower levels of vitamin metabolism, lipid metabolism and detoxification. However, microbiological processes involved in carbohydrate metabolism were more prevalent. At the same time, the oxidative phosphorylation and synthesis of vitamin B are also the characteristics of exclusive breastfeeding (320). At the age of 3 & 6 months, formula-fed infants also have higher levels of SCFAs in their stools, including free amino acids, butyrate, propionate, acetate and 5-amino valerate. Additionally, the breastfed infants had higher levels of lactic acid and FLs in their stools due to higher HMOSs fermentation (323). Therefore, microbiome composition is modulated by the type of feed. The impact of feeding on preterm and term gut microbiota may be potentially beneficial to metabolism, intestinal function and the immune system, along with persisting subject for life (324).

### 5.2. Nutrient profile and processing impact on gut microbiome

Compared to breastfeeding, formula feeding has been linked to a less stable microbiome across time, a remarkably different bacterial composition, and increased bacterial diversity and richness (325–327). In an investigation that operated on 91 infants, the composition of fecal bacteria was more diverse in formula-fed infants. Also, this diversification varies from one formula to another (326). Moreover, the alteration of the microbial profile in the first months of life may lead to overweight development in the infant (328, 329). During the first 18 months, Selma-Royo et al. (330) outlined a lower concentration of *Bifidobacterium* and Bacteroidetes genus in infants with higher Weight/Length and Body Mass Index scores. In another study, infants who were not exclusively breastfed had more Bacteroides count (331). The exact role played by specific bacterial genera or families in weight management is still unclear (309, 332). Overall, these data imply that prior colonization of the gut microbiota is crucial, and it may influence neonatal growth trajectories (333). Moreover, the disruption in gut microbiota (i.e., dysbiosis) has been related to necrotizing enterocolitis and several other chronic illnesses, including inflammatory bowel disease, allergies, obesity, asthma, diabetes, cancer and neurological conditions associated with the gut-brain axis (334–336).

Besides, IF-fed infants have a faster development curve than age-matched breastfed newborns, which is generally associated with advanced adiposity, greater weight gain, and an elevated risk of childhood obesity (337). Piglets raised in containment facilities on bovine-based IF exhibit differences in the mucosal immune system’s three compartments, including more rapid recruitment of antigen presenting cells, lesser regulatory T-cells and increased B-cells, as well as distinctive microbiomes (338, 339). The introduction of IF before the third month of life is associated with a higher risk of rapid growth at six months and increased body mass index in their adulthood. Therefore, the time of formula administration is also a factor in determining health in neonatal and adulthood (340). The consequence of fast gut maturation due to IF feeding is a greater abundance of different *Clostridium* genera, particularly *C. difficile,* with subsequent development of atopy (341). However, only a few research has sought to shed light on the dynamics of the metabolic and functional processes related to the gut microbiota evolution and maturation with impact on long term health, even though many papers have characterized community membership in the gut microbiota (Figure 4).

The macronutrient profile of IFs has the ability to influence the gut microbiome (308). Compared to a casein-predominant IF, a whey-predominant IF resulted in a fecal microbiota that was more similar to that of 2-month-old breastfed infants (342). Nowak-Wegrzyn et al. (86) assessed the safety and clinical hypoallergenicity of a whey-based extensively hydrolyzed formula containing lacto-N-neotetraose and 2′fucosyl-lactose. They suggested that this formula could be recommended for the management of cow’s milk protein allergy in young children and infants. The findings of Rosa et al. (343) indicated that the neonatal diet had an impact on the microbial proteins and bacterial taxa of the gut before weaning. Proteins like lactoferrin may also serve as prebiotics and affect the composition of gut microbiota. Manzoni (344) documented the *in vivo* effect of lactoferrin on the diversity and growth of intestinal microbiota. The advantage of lactoferrin via the microbiota might involve several processes, including intestinal cell differentiation and maturation, energy supply for growth of the intestine and gut-brain-microbiota axis signaling for the development of nerve fibers (345–347). Gomez-Gallego et al. (348) investigated weaned pups (*n* = 12) fed with low-concentration polyamines-enriched IF (putrescine, spermidine & spermine). The microbiota changes were higher on supplementation, followed by increased levels of *Akkermansia*-like bacteria, *Bifidobacterium* group and *Lactobacillus*–*Enterococcus* group bacteria.

The triglyceride structure may potentially have an impact on bacteria in the gut. Compared to infants receiving a low-palmitate IF, high-palmitate IF increased *Bifidobacterium* and *Lactobacillus* count in 6-week-old infants’ faces at quantities similar to that of breastfed children (349). In accordance with a study carried out on germ-free female and male pups (*n* = 36), the establishment of the gut microbiota may be modulated by the differences in phospholipids types and the fatty acids profile. The results suggest that the emulsifiers and fat type used in IF may affect gut flora setup differently in formula-fed infants (350). 1,3-olein-2-palmitin is a triglyceride used in the supplementation of IF, demonstrated a slight increase of SCFA content and an abundance of gut *bifidobacteria* (351). In 28-day-old piglets, the addition of MFGM alone did not affect the gut microbiota composition compared to standard IFs. When compared to piglets fed on a regular vegetable-oil IF, there was a rise in *Proteobacteria* and fall in *Firmicutes* phyla showing the impact of MFGM and dairy lipids. This incorporation of MFGM and milk fat in IF modifies fecal microbiota composition, the process of gut immunity development and protein digestion (352).

Interventions containing exogenous microbes have recently gained attention in the modulation of gut microbiota (353, 354). Lemaire et al. (355) investigated the long-term effects of *Lactobacillus fermentum* and dairy lipids on gut microbiota, host entero-insular axis & metabolism. The intervention specifically lowered the fecal content of lysine and 2-oxoglutarate. It also increased SCFA content and had a beneficial impact on endocrine function. In line with this, *Bifidobacterium* strains work as ecosystem engineers in extremely preterm infants, speeding up the development of their microbiomes and having an impact on their immune systems (356). Since *Limosilactobacillus reuteri* has been found both in human gut and breast milk, Alliet et al. (357) evaluated the efficacy and safety of IF supplemented with 2′-fucosyllactose & *L. reuteri*. Results suggest that doing so supports age-appropriate growth, is well-tolerated and may play a role in shifting the gut microbial pattern toward that of breastfed infants. Meanwhile, by generating SCFAs, probiotic metabolites can support an anti-inflammatory effect (358, 359). For example, butyrate reduces the expression of the pro-inflammatory cytokines and enhances the expression of the anti-inflammatory cytokines suggesting that it is a key negative regulator of inflammation. Acetate is another abundantly produced SCFA that exerts an anti-inflammatory effect on receptors expressed in peripheral blood cells and adipose tissue (360).

Again, various types of prebiotic substrates might perhaps act diversely on intestinal bacteria (316). In the colon, prebiotics reach intact and serve as substrates for bacterial growth. Therefore, the Bifidobacterium count and metabolic activity of gut microbiota of infants supplemented with the prebiotics is comparable to that of breastfed infants (361). Furthermore, the infants fed with 2′-FL & LNnT-supplemented IF had fecal microbiota similar to breastfed infants in terms of overall genus composition, microbial diversity and abundance of major genera (153, 362, 363). Infants fed on IF supplemented with 2′-FL expressed lower levels of plasma inflammatory cytokine profile (IL1- α, IL1-β, TNFα & IL 6), which resembles that of breastfed infant group (364). Following this, the incorporation of a prebiotic mix in IF resulted in an elevated concentration of stool secretory IgA and reduced infection rates. Therefore, prebiotics are also indicated to have an influence on intestinal immune development (361). Lagkouvardos et al. (365) demonstrated that synbiotic intervention containing GOS and *Limosilactobacillus fermentum* influenced milieu parameters and fecal microbiota at an early age, sharing fewer similarities to breastfed infants. The use of a probiotic-supplemented IF, notably with *S. thermophilus* and *B. lactis*, has displayed a lower incidence of antibiotic-associated diarrhea. Due to the heterogeneity and limited numbers of studies, it is challenging to draw more robust conclusions about the exact intervention of both pro- and prebiotics for supplementation in IF to modulate the gut microbiome.

Likewise, some food additives may also disrupt gut homeostasis and contribute to inflammatory responses related to tissue damage (366). Several studies in animal models, as reviewed by Rinninella et al. (367), reported that higher salt doses are associated with changes in bacterial group abundance. These results depicted decreased group abundance in *Oscillibacter*, *Lactobacillus* spp., *Pseudoflavonifractor*, *Rothia*, *Clostridium* and *Johnsonella*; and increased group abundance in *Ruminococcus*, *Parasutterella* spp., *Lachnospiraceae*, *Erwinia* genus, *Corynebacteriaceae* and *Christensenellaceae*. Furthermore, work on the effect of vitamin D supplementation in infant gut microbiota (age 3–6 months) demonstrated that, Vit. D was associated with decreased *Lactococcus* and increased *Lachnobacterium*. These studies on Vit. D also suggested the possibilities of correlations in the incidence and prevalence of allergies/asthma with long-term immune system implications (368). The B vitamins are biosynthesized by the cooperation of several phyla of gut bacteria, including *Bacteroidetes*, *Proteobacteria* and *Fusobacteria* (369). Emulsifiers as an additive lower gut microbial diversity, i.e., increasing *Verrumicrobia* and decreasing *Bacteroides* abundance. These alterations in gut microbiota led to dysbiosis and promoted colitis, chronic gut inflammation and metabolic syndrome (370). Therefore, all such considerations must be considered while designing infant formula mimicking human milk.

Processing causes significant alteration in the composition and structure of different components present in food. Because of heat treatment, the Maillard reaction occurs, resulting in the formation of melanoidins which possess prebiotic role and, in turn, affect gut microbiota composition. For example, melanoidin-enriched malts were fed to mice (*n* = 75) and relative reduction in pathogenic bacterial groups, namely *Dorea*, *Alistipe*, and *Oscillibacter*; and an increase in beneficial *Lactobacillus*, *Akkermansia*, *Parasutterella*, *Barnesiella*, *Bifidobacterium* were observed (371). In addition, prior to spray drying, heat treatment of milk was shown to improve the residual casein immunoreactivity significantly (372). Ye et al. (239) demonstrated that storage and UHT treatment of ready-to-feed liquid IF affects *in vitro* digestibility and release of bioactive peptides, principally due to the induction of protein aggregation and structural changes. These peptides are more readily absorbed, improving the digestibility. The intense cooking technologies sometimes increase the abundance of beneficial microbes like *Bifidobacterium* spp. and *Ruminococcus* spp. (373). Reviewing different studies, we have concluded that there is still lack of more vigorous research about processing of IF and their impact on gut microbiota.

The formation of the gut microbiota is important, but since it is so complex, it is still not entirely understood how various pathways affect the biology of the infant (308). In order to investigate the early nutritional effect in the long term and to control some confounding factors discovered in human studies, preclinical models like animals such as neonatal piglets and nonhuman primates are found to be excellent for such studies (374–376). Alongside, targeting microbiome reprogramming for the treatment and prevention of metabolic illnesses in the future will be made possible by a comprehensive knowledge of relationships between gut microbiome establishment, metabolism and human health (377). Correlations between particular bacteria and certain macronutrients have been documented; however, more research is required to fully comprehend these associations and assess their long-term health effects (378).

## 6. Conclusion

Infant Formula consumption is increasing enormously in this modern era, creating a striving need to develop formulas that fulfil the growth and developmental requirements of infants who cannot breastfeed. Researchers and scientists are working on adding different components and applying the latest technologies to formulate products like human milk in many possible ways. The paramount interest is shifting toward adding functional bioactive compounds that can promote health outcomes other than growth, namely cognition, immunity, and temperament. There are numerous technologies involved when it comes to infant formula processing. Most production processes involve heat for microbial inactivation and powder production, causing heat-induced deteriorative changes in IF. Many non-thermal technologies have been utilized at different stages of IF manufacturing to overcome the drawbacks of nutritive loss from heat treatments. In terms of IF action in the gut, the development of an infant’s gut microbiota plays a pivotal role, but because of its complexity, underlying pathways having an impact on an infant’s gut biology remain unknown. Additionally, scientists have reported the beneficial effect of milk derived constituents on gut microbiota development which is related to the development and maintenance of metabolic health and immunity. Yet due to the lack of an exact relationship between formula feeding, gut microbiota establishment and its long-term health implications, greater in-depth and extensive research in these fields are of utmost importance. Likewise, a sound understanding of the physiological properties of IF during storage, handling and transportation must be evaluated. On top of that, ensuring sustainability and reducing production costs are also crucial for Infant formula success.

## Author contributions

SB, VP, and DB: conceptualization. SB and BB: methodology. SB, VP, and SK: data curation. SB, VP, SY, ZA-Z, and HR: writing and original draft preparation. DB, BB, SK, PK, and VK: review and editing of the manuscript. SB, PK, VK, and ZA-Z: visualization and illustrations. VP, DB, and SY: supervision. All authors have read and agreed to the published version of the manuscript.

## Funding

VP acknowledges the IoE Scheme research grant from Banaras Hindu University, Varanasi, India.

## Conflict of interest

The authors declare that the research was conducted in the absence of any commercial or financial relationships that could be construed as a potential conflict of interest.

## Publisher’s note

All claims expressed in this article are solely those of the authors and do not necessarily represent those of their affiliated organizations, or those of the publisher, the editors and the reviewers. Any product that may be evaluated in this article, or claim that may be made by its manufacturer, is not guaranteed or endorsed by the publisher.
