# Supplementary figures and images for: A comprehensive review on infant formula: nutritional and functional constituents, recent trends in processing and its impact on infants’ gut microbiota
Source: Front Nutr. 2023 Jun 21;10:1194679. doi: 10.3389/fnut.2023.1194679 (PMC10320619; doi:10.3389/fnut.2023.1194679)

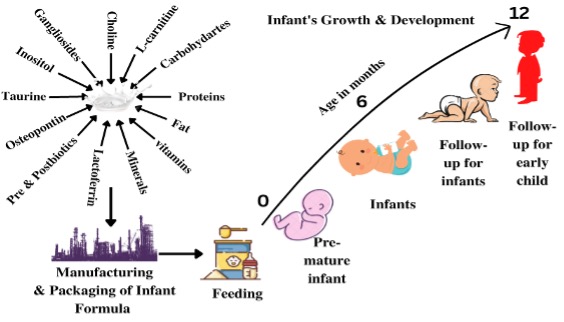

Supplement: Supplementary file 1 [file Image_1.JPEG]
